# Engineering catalytic defects via molecular imprinting for high energy Li-S pouch cells

**DOI:** 10.1093/nsr/nwae190

**Published:** 2024-05-31

**Authors:** Yufei Zhao, Chuannan Geng, Li Wang, Yun Cao, Haotian Yang, Linkai Peng, Xin Jiang, Yong Guo, Xiaolin Ye, Wei Lv, Quan-Hong Yang

**Affiliations:** Shenzhen Geim Graphene Center, Engineering Laboratory for Functionalized Carbon Materials, Tsinghua Shenzhen International Graduate School, Tsinghua University, Shenzhen 518055, China; Nanoyang Group, Tianjin Key Laboratory of Advanced Carbon and Electrochemical Energy Storage, School of Chemical Engineering and Technology, and Collaborative Innovation Center of Chemical Science and Engineering (Tianjin), Tianjin University, Tianjin 300072, China; Joint School of National University of Singapore and Tianjin University, International Campus of Tianjin University, Fuzhou 350207, China; Shenzhen Geim Graphene Center, Engineering Laboratory for Functionalized Carbon Materials, Tsinghua Shenzhen International Graduate School, Tsinghua University, Shenzhen 518055, China; Nanoyang Group, Tianjin Key Laboratory of Advanced Carbon and Electrochemical Energy Storage, School of Chemical Engineering and Technology, and Collaborative Innovation Center of Chemical Science and Engineering (Tianjin), Tianjin University, Tianjin 300072, China; Nanoyang Group, Tianjin Key Laboratory of Advanced Carbon and Electrochemical Energy Storage, School of Chemical Engineering and Technology, and Collaborative Innovation Center of Chemical Science and Engineering (Tianjin), Tianjin University, Tianjin 300072, China; Joint School of National University of Singapore and Tianjin University, International Campus of Tianjin University, Fuzhou 350207, China; Shenzhen Geim Graphene Center, Engineering Laboratory for Functionalized Carbon Materials, Tsinghua Shenzhen International Graduate School, Tsinghua University, Shenzhen 518055, China; Shenzhen Geim Graphene Center, Engineering Laboratory for Functionalized Carbon Materials, Tsinghua Shenzhen International Graduate School, Tsinghua University, Shenzhen 518055, China; Nanoyang Group, Tianjin Key Laboratory of Advanced Carbon and Electrochemical Energy Storage, School of Chemical Engineering and Technology, and Collaborative Innovation Center of Chemical Science and Engineering (Tianjin), Tianjin University, Tianjin 300072, China; Joint School of National University of Singapore and Tianjin University, International Campus of Tianjin University, Fuzhou 350207, China; Shenzhen Geim Graphene Center, Engineering Laboratory for Functionalized Carbon Materials, Tsinghua Shenzhen International Graduate School, Tsinghua University, Shenzhen 518055, China; Nanoyang Group, Tianjin Key Laboratory of Advanced Carbon and Electrochemical Energy Storage, School of Chemical Engineering and Technology, and Collaborative Innovation Center of Chemical Science and Engineering (Tianjin), Tianjin University, Tianjin 300072, China; Nanoyang Group, Tianjin Key Laboratory of Advanced Carbon and Electrochemical Energy Storage, School of Chemical Engineering and Technology, and Collaborative Innovation Center of Chemical Science and Engineering (Tianjin), Tianjin University, Tianjin 300072, China; Nanoyang Group, Tianjin Key Laboratory of Advanced Carbon and Electrochemical Energy Storage, School of Chemical Engineering and Technology, and Collaborative Innovation Center of Chemical Science and Engineering (Tianjin), Tianjin University, Tianjin 300072, China; Shenzhen Geim Graphene Center, Engineering Laboratory for Functionalized Carbon Materials, Tsinghua Shenzhen International Graduate School, Tsinghua University, Shenzhen 518055, China; Nanoyang Group, Tianjin Key Laboratory of Advanced Carbon and Electrochemical Energy Storage, School of Chemical Engineering and Technology, and Collaborative Innovation Center of Chemical Science and Engineering (Tianjin), Tianjin University, Tianjin 300072, China; Joint School of National University of Singapore and Tianjin University, International Campus of Tianjin University, Fuzhou 350207, China

**Keywords:** lithium-sulfur battery, catalysts, defects, energy density, metal sulfide

## Abstract

Heterogeneous catalysis promises to accelerate sulfur-involved conversion reactions in lithium-sulfur batteries. Solid-state Li_2_S dissociation remains as the rate-limiting step because of the weakly matched solid-solid electrocatalysis interfaces. We propose an electrochemically molecular-imprinting strategy to have a metal sulfide (MS) catalyst with imprinted defects in positions from which the pre-implanted Li_2_S has been electrochemically removed. Such tailor-made defects enable the catalyst to bind exclusively to Li atoms in Li_2_S reactant and elongate the Li–S bond, thus decreasing the reaction energy barrier during charging. The imprinted Ni_3_S_2_ catalyst shows the best activity due to the highest defect concentration among the MS catalysts examined. The Li_2_S oxidation potential is substantially reduced to 2.34 V from 2.96 V for the counterpart free of imprinted vacancies, and an Ah-level pouch cell is realized with excellent cycling performance. With a lean electrolyte/sulfur ratio of 1.80 μL mg_S_^–1^, the cell achieves a benchmarkedly high energy density beyond 500 Wh kg^–1^.

## Teaser

Electrochemical molecular imprinting is proposed to custom-tailor defects for catalytic conversion of solid lithium sulfide towards high-energy lithium-sulfur batteries.

## INTRODUCTION

Metal-sulfur batteries with high energy densities have received much attention in recent years. The lithium-sulfur (Li-S) battery is a typical example featuring a high capacity caused by the conversion between sulfur and lithium sulfide (Li_2_S) in the cathode [1–5]. However, this conversion also results in rapid sulfur loss and poor cycling performance because of the dissolution and shuttling of dissolved intermediate lithium polysulfides (LiPSs). It is widely accepted that the LiPS-to-Li_2_S conversion is rate-determining in the sulfur reduction reaction (SRR), which causes the accumulation of LiPSs in the electrolyte and severe shuttle effects. Heterogeneous electrocatalysis has been developed to improve the reaction kinetics of the sulfur cathode [1,5–7]. Various solid catalysts, including metal oxides [8], sulfides [9], selenides [10], phosphides [11], nitrides [12], and single-atom catalysts [13], and even doped graphenes [1,14], have been reported in a large number of studies. Among them, metal sulfides (MSs) have shown excellent adsorption and catalytic ability for the sulfur species in Li-S batteries because of their strong interactions with LiPSs and Li_2_S through metal-sulfur and Li-sulfur bonds [9]. Although an apparent improvement in performance is achieved, the maladies of fast capacity decay for the battery are still difficult to be fundamentally cured. The main reason for this is the large reaction barrier of Li_2_S-to-LiPS conversion in the sulfur evolution reaction (SER) during charging, which causes incomplete conversion of Li_2_S and electrode passivation in the overall discharge-charge process, particularly in practical use of lean electrolyte and high sulfur loading [15,16]. Heterogeneous catalysis is normally useless when facing such a problem due to the poor affinity between the solid catalyst and solid Li_2_S, which severely limits the reversible conversion of Li_2_S [17]. This is usually overlooked, while, for a battery, the Li_2_S-to-LiPS conversion intrinsically is the primary rate-limiting step not only for SRR but also for the whole reversible sulfur conversion.

**Figure 1.**
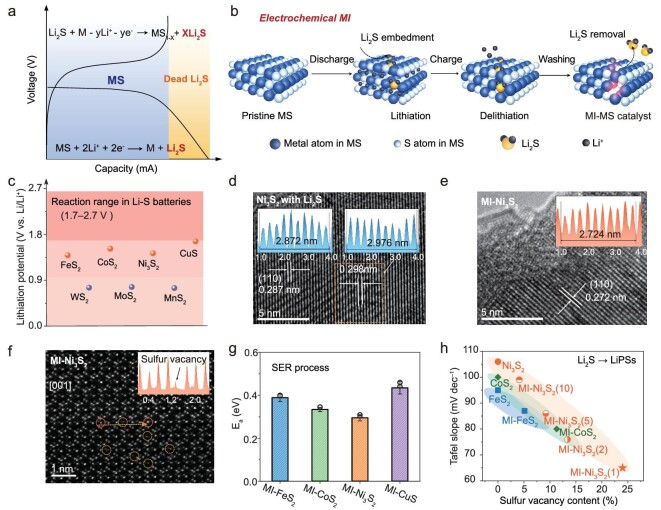
Principles of electrochemical molecular imprinting. (a) Discharge and charge curves for MSs. (b) Illustration of electrochemical MI strategy. (c) Lithiation potential distribution for different MSs. (d) and (e) HRTEM images of Ni_3_S_2_ (d) after delithiation, and (e) after removing Li_2_S. (f) Spherical aberration-corrected HAADF-STEM image in the [001] direction of MI-Ni_3_S_2_. (g) Apparent activation energies of the SER process for MI-MS catalysts calculated from the CV profiles. The data are presented as mean values, and error bars are given. (h) The relationship between the Tafel slope, obtained using the CV profiles of the various catalysts, and the sulfur vacancy contents from ICP-MS results, the MI-Ni_3_S_2_(1–10) represents the material obtained from Ni_3_S_2_ batteries with different cycle numbers.

To achieve the benchmarked energy density of 500 Wh kg^–1^, it is critically important to overcome the bottleneck of Li_2_S conversion within heterogeneous catalysis. Molecular imprinting (MI) seems like a ‘key’ solution to the poor affinity of Li_2_S since, in heterogeneous catalysis, by implanting-removing targeted reactant molecules, the molecularly imprinted sites formed on the catalyst surface enable the catalyst to selectively and strongly bind to the reactants in catalysis, substantially increasing catalytic efficiency [18,19]. Unfortunately, it is challenging to composite and interact with Li_2_S within most heterogeneous catalysts because of the Li_2_S instability in air and the lack of intermediate binding sites. MSs have caught our attention because they can be lithiated to form mixed phases of metal and Li_2_S during discharge in Li-ion batteries. As shown in Fig. 1a, MSs have irreversible capacities in charging, which means that some Li_2_S cannot be converted and can be implanted into the MS lattice. This phenomenon allows us to imprint molecular Li_2_S vacancies in the bulk of MSs by removing the Li_2_S with a polar solvent washing process (Fig. 1b) [20].

Following this, we herein report an electrochemically MI strategy to imprint the tailored defects in the bulk of MS catalysts (denoted as MI-MSs), which leads to an uneven charge distribution of surface sulfur atoms in MSs, enabling their selective binding to Li atoms in the Li_2_S reactant. The enhanced interaction helps form molecularly-bound solid-solid interfaces and lengthens the Li-S bond in catalysis, significantly decreasing the Li_2_S dissociation energy barrier. MI-Ni_3_S_2_ has the best catalytic activity for Li_2_S oxidation due to its highest vacancy content among the MS catalysts investigated. The apparent activation energy (*E*_a_) of Li_2_S-to-LiPS conversion decreases from 0.44 (with Ni_3_S_2_, free of imprinted vacancies) to 0.35 eV (with MI-Ni_3_S_2_), and the Li_2_S oxidation potential decreases from 2.96 to 2.34 V, which is very close to the thermodynamically predicted oxidation potential of Li_2_S (∼2.3 V [21]) and much lower than those described for most of the reported nanostructured Li_2_S cathodes (2.5–3.5 V [16,22–25]). The tested coin-cell Li-S battery using this MI-Ni_3_S_2_ catalyst had a high capacity of 655 mAh g^−1^ at 5C and good cycling stability over 1000 cycles with a low decay rate of 0.05% at 1C; with a high sulfur loading (11.2 mg cm^–2^) and a low electrolyte/sulfur ratio (E/S = 5.6 μL mg_S_^–1^), a high areal capacity of 6.65 mAh cm^–2^ was still achieved after 100 cycles. An assembled Ah-level pouch cell with 311 Wh kg^−1^ could stably work over 50 cycles and the largest-capacity cell features an energy density of up to 502 Wh kg^−1^ at an ultralow E/S ratio (1.80 μL mg_S_^−1^). Our work provides an effective way and, more importantly, a rationale to synthesize a practical catalyst with a well-managed solid-solid interfacing not limited to high-energy sulfur-based batteries.

## RESULTS AND DISCUSSION

### Electrochemically MI strategy

Various MSs (FeS_2_, CoS_2_, Ni_3_S_2_, CuS, MoS_2_, WS_2_, and MnS_2_) were employed to verify the electrochemically MI strategy. In a half cell with a Li metal counter electrode [18], Fig. 1c, and Fig. S1a and b show the lithiation platforms ∼0.5–1.6 V, beyond the voltage window of Li-S batteries (1.7–2.7 V). For the MSs with the nonlayered structure (FeS_2_, CoS_2_, Ni_3_S_2_, and CuS_2_), the conversion reactions (${\mathrm{MS + }}2{\mathrm{L}}{{{\mathrm{i}}}^ + } + 2{{{\mathrm{e}}}^ - } \to {\mathrm{L}}{{{\mathrm{i}}}_2}{\mathrm{S}} + {\mathrm{M}}$) occur at lithiation potentials of 1.39, 1.51, 1.43, and 1.65 V, respectively. They show partially irreversible delithiation because dead Li_2_S is retained in the MSs lattice, resulting in a low initial coulombic efficiency (CE) (Fig. S2). The Li_2_S signals were observed in Raman spectra after the first cycle (Fig. S3a–c). Taking Ni_3_S_2_ as an example, the high-resolution transmission electron microscope (HRTEM) image (Fig. 1d) shows that the (110) spacing of Ni_3_S_2_ increases from 2.87 Å to 2.98 Å, which is ascribed to residual Li_2_S in the Ni_3_S_2_ lattice. In addition, the titration experiments by Ag(NO)_3_ also strongly proves the existence of Li_2_S (Fig. S4a and Supplementary discussion). However, no Li_2_S crystal was observed in the HRTEM image (Fig. S4b), and in the XRD pattern of Ni_3_S_2_ electrode after one cycle (Fig. S4c), neither characteristic peak of Li_2_S was found. These results indicate that the Li_2_S exists in an amorphous state. After removing the Li_2_S by alcohol washing, the obtained samples are denoted MI-MSs. A CuS electrode shows good reversibility (Fig. S1a and Fig. S3d) and is therefore unsuitable for use in producing the MI-MSs. X-ray diffraction (XRD) pattern results show that the MI-MSs have similar characteristic diffraction peaks to the original MSs (Fig. S5), suggesting the crystal structure was well maintained after removing Li_2_S. MSs with the layer structure (MoS_2_, WS_2_, and MnS_2_) have low lithiation potentials of 0.754, 0.765, and 0.742 V (Fig. 1c and Fig. S1b) and undergo irreversible structural collapse and even amorphization as shown in XRD patterns in Fig. S6 [26]. They are therefore not suitable for electrochemical MI.

The HRTEM image shows a narrower (110) spacing (2.72 Å) for MI-Ni_3_S_2_ than for pure Ni_3_S_2_ (2.87 Å) due to structural distortion and reorganization (Fig. 1e) [27]. Spherical aberration-corrected high-angle annular dark-field scanning transmission electron microscope (HAADF-STEM) imaging suggests the typical rhombohedral phase of MI-Ni_3_S_2_ (Fig. S7). Sulfur vacancies are observed by comparison of extraction intensities in adjacent crystal regions (the yellow arrow region in Fig. 1f). MI-Ni_3_S_2_ has a similar XRD pattern to the original Ni_3_S_2_ (Fig. S8a), but the (110) peak (31.01°) of MI-Ni_3_S_2_ moves to a slightly higher angle (31.27°) due to defect-induced structural distortion (Fig. S8b), in agreement with the HRTEM image. In addition, ethanol washing does not affect the Ni_3_S_2_ structure, as confirmed by the XRD pattern (Fig. S8a). X-ray photoelectron spectra (XPS) show the phase and composition changes from Ni_3_S_2_ to MI-Ni_3_S_2_ [28]. The Ni 2p peaks in MI-Ni_3_S_2_ shift to lower binding energies, and a pair of peaks ∼852.8 eV corresponding to Ni^0^ are observed, which is attributed to the partial reduction of Ni^2+^ due to the loss of S (Fig. S9a) [29,30]. A similar phenomenon is also observed in MI-FeS_2_ and MI-CoS_2_ (Fig. S10). The inductively coupled plasma mass spectrometry (ICP-MS) results also show that the amount of sulfur loss in MI-MS correlates negatively with the initial CE (Table S1 and Fig. S11).

Li-S cells with CNT/S cathodes containing various MI-MSs as catalysts were assembled to show their activity in sulfur redox reactions. In the cyclic voltammetry (CV) profiles (Fig. S12), the electrodes containing MI-FeS_2_, MI-CoS_2_, MI-Ni_3_S_2_, and MI-CuS have a higher peak intensity than those with the original counterparts, suggesting increased sulfur redox conversion. Note that the electrodes with MI-MSs have lower oxidation onset potentials (peak A1, at ∼2.3 V), indicating the improved reaction kinetics for Li_2_S-to-LiPSs conversion in the SER (Fig. S13). Temperature-dependent CV measurements also show the oxidation kinetics of different catalysts by calculating the *E*_a_ from the A1 peak (Fig. S14) [31]. The *E*_a_ values follow the order MI-Ni_3_S_2_ (0.29 eV) < MI-CoS_2_ (0.33 eV) < MI-FeS_2_ (0.39 eV) < MI-CuS (0.43 eV) (Fig. 1g), and are proportional to the loss of sulfur content in preparing the MI-MS (Table S1). We also prepared MI-Ni_3_S_2_ with different numbers (1–10) of lithiation/delithiation cycles to quantify the relationship between defect structure and Li_2_S affinity (Fig. S15). The results show that the sulfur vacancy content is inversely proportional to the Tafel slope obtained from the oxidation peak of Li_2_S-to-LiPSs in the CV profiles (Fig. 1h, Fig. S16, and Tables S1 and S2), which indicates the defect structure tailored for Li_2_S in MSs can significantly promote Li_2_S conversion. Among the various MSs investigated, MI-Ni_3_S_2_(1) has the best catalytic ability for Li_2_S conversion with the smallest Tafel slope, and is used in the following discussion to show the roles and mechanisms of MI catalysts.

### Function of MI for Li_2_S catalysis

**Figure 2.**
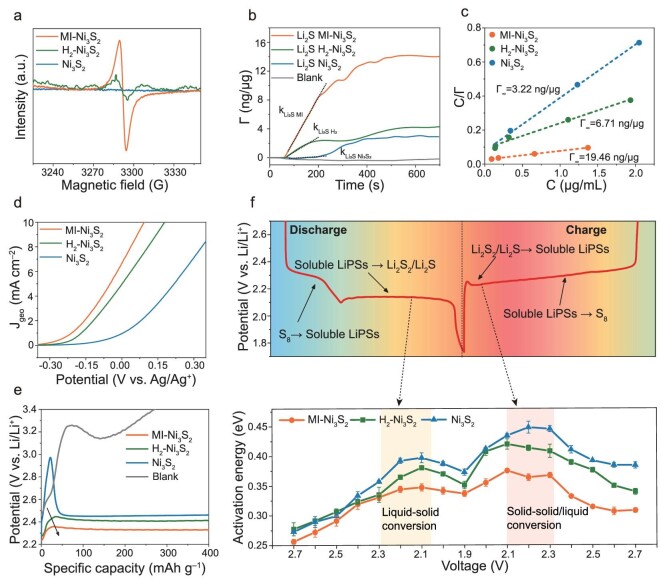
Roles of the molecularly imprinted catalyst. (a) EPR spectra of MI-Ni_3_S_2_, H_2_-Ni_3_S_2_, and Ni_3_S_2_. (b) Mass changes of MI-Ni_3_S_2_, H_2_-Ni_3_S_2_, and Ni_3_S_2_ for Li_2_S adsorption at 0.03 mM. (c) Adsorption curves for MI-Ni_3_S_2_, H_2_-Ni_3_S_2_, and Ni_3_S_2_ using the Langmuir adsorption model. (d) Linear sweep voltammetry (LSV) curves of MI-Ni_3_S_2_, H_2_-Ni_3_S_2_, and Ni_3_S_2_ electrodes for SER at 10 mV s^−1^. (e) Charge voltage profiles of Li_2_S cathodes without and with different catalysts in the first cycle. (f) Galvanostatic discharge-charge profiles of a Li-S cell, and the activation energies (*E*_a_) for MI-Ni_3_S_2_, H_2_-Ni_3_S_2_, and Ni_3_S_2_ at various given voltages. The data are presented as mean values, and the error bars are shown.

To distinguish the difference between an MI-catalyst and the usually reported catalysts with sulfur vacancies, Ni_3_S_2_ was treated in an Ar/H_2_ (5%) atmosphere to introduce vacancies [32]. The obtained sample is denoted H_2_-Ni_3_S_2_, and its XRD (110) peak shifted to a higher angle (31.14°) due to the smaller lattice produced by the vacancies (Fig. S8b). ICP-MS results show similar sulfur contents for MI-Ni_3_S_2_ and H_2_-Ni_3_S_2_ (24.5 at% and 22.6 at%). The electron paramagnetic resonance (EPR) results for MI-Ni_3_S_2_ and H_2_-Ni_3_S_2_ in Fig. 2a show a pair of opposite peaks at g = 2.003 compared with Ni_3_S_2_ due to the unpaired electrons induced by the sulfur vacancies. The higher peak intensity for MI-Ni_3_S_2_ is due to the higher S loss in the bulk lattice [33]. In addition, in Fig. S9b, the S 2p XPS spectra showing the higher binding energy peak (164.0 eV) is attributed to incompletely coordinated surface sulfur (S_surf_), and the lower binding energy peak (162.2 eV) to lattice sulfur (S_latt_) fully bonded to Ni, referring to the correlation analysis of oxygen vacancies [34,35]. The S_surf_ peaks of MI-Ni_3_S_2_ and H_2_-Ni_3_S_2_ show a negative shift compared to that of Ni_3_S_2_, suggesting the formation of surface defects in both samples [36]. However, only MI-Ni_3_S_2_ has a negative shift for the S_latt_ peak and a significant decrease in the S_latt_ peak ratio due to the formation of MI sites in the bulk [37,38].

To verify the selective binding ability of Li_2_S derived from the MI [39], a quartz crystal microbalance (QCM) was used to characterize the adsorption rate and adsorption capacity of Li_2_S dissolved in methanol (0.03 mM) on the above several catalysts [40]. As shown in Fig. 2b, the time-mass change profiles show the rate of mass increase (k_Li2S MI- Ni3S2_) on MI-Ni_3_S_2_ was ∼27.8 and 4.1 times higher than those on Ni_3_S_2_ (k_Li2S Ni3S2_) and H_2_-Ni_3_S_2_ (k_Li2S H2-Ni3S2_) in the initial stage. In addition, H_2_-Ni_3_S_2_ and Ni_3_S_2_ showed almost equal adsorption capacity, much lower than MI-Ni_3_S_2_, which are no specificity for Li_2_S. Li_2_S solutions with different concentrations were used to quantitatively determine the adsorption capacity (Fig. S17). The MI-MS adsorption of Li_2_S was fitted by the Langmuir adsorption model in Eqn. (1) (Fig. 2c):


(1) \begin{eqnarray*}
\frac{c}{\Gamma } = \frac{1}{{{{\Gamma }_\infty }k}} + \frac{1}{{{{\Gamma }_\infty }}}c,\
\end{eqnarray*}

where *Γ* is the adsorption amount; *Γ_∞_* is the saturation adsorption capacity; *c* is the concentration of the solution in the adsorption equilibrium, and *k* is constant [41]. The saturation adsorption capacity of MI-Ni_3_S_2_ for Li_2_S is 19.22 ng (Li_2_S)/μg (catalyst), much higher than those for H_2_-Ni_3_S_2_ (6.71) and Ni_3_S_2_ (3.22), demonstrating the much stronger binding to Li_2_S and the selective adsorption of Li_2_S by electrochemical MI strategy compared with common sulfur vacancy (Supplementary discussion and Table S3).

Linear sweep voltammetry (LSV) measurements with a rotating disk electrode (RDE) were also conducted in a 0.1 M Li_2_S/methanol solution with a sweep rate of 10 mV s^−1^ in the voltage range from −1 to 1 V (*vs.* Ag/AgCl). MI-Ni_3_S_2_ shows a higher onset potential than Ni_3_S_2_ and H_2_-Ni_3_S_2_ (Fig. 2d). The corresponding Tafel plots (Fig. S18) implying an oxidation reaction from insoluble Li_2_S to soluble LiPSs also show a smaller slope of 195 mV/dec for MI-Ni_3_S_2_ than for H_2_-Ni_3_S_2_ (229 mV/dec) and Ni_3_S_2_ (283 mV/dec). To directly show the catalytic ability of MI-Ni_3_S_2_ for Li_2_S oxidation, the activation potential for a commercial Li_2_S cathode in the first cycle was also investigated [16]. The Li_2_S cathode without the catalysts (blank sample) has a high oxidation potential (3.14 V), as shown in Fig. 2e, which decreases to 2.34 V after introducing MI-Ni_3_S_2_, much lower than those with Ni_3_S_2_ (2.96 V) and H_2_-Ni_3_S_2_ (2.45 V), proving its good catalytic ability, outperforming most of the reported Li_2_S cathodes (Fig. S19).

Figure 2f shows typical charge-discharge curves for SRR and SER. The *E*_a_ at different potentials were calculated by measuring the charge transfer resistance at various temperatures using electrochemical impedance spectroscopy (EIS) and fitting the results to the Arrhenius equation (Figs S20–S22) [14]. The simplified-contact Randle-equivalent circuit contains the resistances of Li^+^ migration in the interfaces between the electrodes and electrolyte (*R*_surf_) and the charge transfer process (*R*_ct_) (Fig. S23) [14]. The logarithmic values of the reciprocal of the charge transfer resistance obey a linear relationship with the reciprocal of the absolute temperature (Fig. S24). The low *E*_a_ at 2.7–2.3 V corresponds to the fast conversion from S_8_ to long-chain LiPSs (Fig. 2f). A sudden increase of *E*_a_ at a voltage of 2.2–2.0 V is shown to be due to the rate-limiting step in SRR, i.e. LiPSs-to-Li_2_S conversion, in good agreement with the literature [31]. The cell with MI-Ni_3_S_2_ has a much lower *E*_a_ (∼0.34 eV) in this range (2.2–2.0 V) than the other two cells, ascribed to the better adsorption and catalytic ability of the defective structure. Note that in the SER for the oxidation of Li_2_S, Ni_3_S_2_ and H_2_-Ni_3_S_2_ have extremely high respective *E*_a_ values of 0.46 eV and 0.42 eV at 2.2–2.3 V, which are for Li_2_S dissociation to LiPSs. *E*_a_ then drops significantly to 0.41 eV (Ni_3_S_2_) at 2.4 V because the LiPSs formed in order to promote the oxidation of Li_2_S through comproportionation [42]. These results suggest the oxidation of Li_2_S is the rate-limiting step in Li-S battery electrochemistry. In contrast, the battery with MI-Ni_3_S_2_ has a much lower *E*_a_ of 0.36 eV at 2.3 V in the SER, which remains stable (∼0.30 eV) throughout charging, proving its excellent catalytic activity for Li_2_S dissociation and LiPS oxidation.

**Figure 3.**
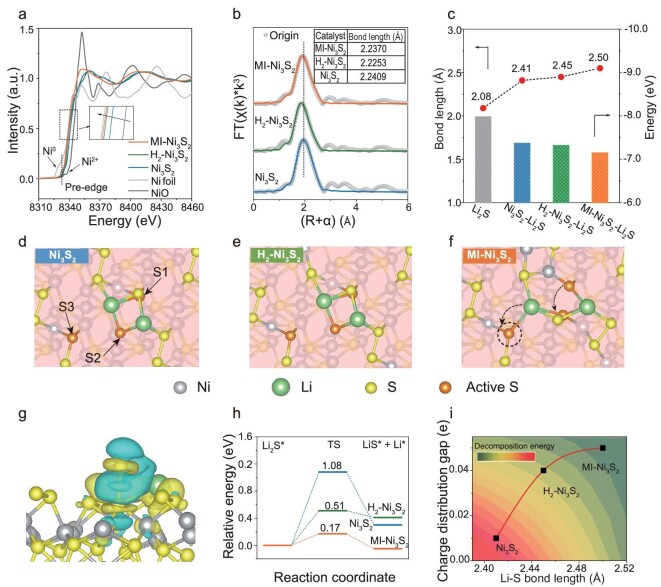
Mechanism of the MI-Ni_3_S_2_ in SER. (a) Normalized XANES spectra of the Ni K-edge. The inset is a magnification of the edge shift with a Ni foil, NiO, and various catalysts. (b) Fourier transform (FT) k3-weighted χ(k)-function of the EXAFS spectra at the Ni K-edge for MI-Ni_3_S_2_, H_2_-Ni_3_S_2_, and Ni_3_S_2_, and their fitted lines. The inset is the Ni-S bond length information from fitted results. (c) Li-S bond lengths and the energies of Li_2_S before and after adsorption on the pristine Ni_3_S_2_, H_2_-Ni_3_S_2_, and MI-Ni_3_S_2_ (110) slabs. (d–f) The optimized adsorption configurations of Li_2_S on Ni_3_S_2_, H_2_-Ni_3_S_2_, and MI-Ni_3_S_2_. (g) The charge density difference of Ni_3_S_2_-Li_2_S. The accumulation and loss of charge are represented by yellow and cyan regions (Isosurface value: 0.02 e Å^−3^), respectively. (h) Energy profiles for the decomposition of a Li_2_S cluster on Ni_3_S_2_, H_2_-Ni_3_S_2_, and MI-Ni_3_S_2_. (i) The correlation between the charge distribution gap (S1, S2) on various catalysts with Li-S bond length of adsorbed Li_2_S.

X-ray absorption spectroscopy at the Ni K-edge, including near edge structure (XANES) and extended fine structure (EXAFS), was used to study the valence state of Ni and local atomic structure. Normalized XANES spectroscopy of the Ni K-edge in all samples shows a pre-edge feature ∼8332 eV, similar to that of standard crystalline Ni_3_S_2_ (Fig. 3a). However, the edge shifts closer to that for a Ni foil for MI-Ni_3_S_2_ and H_2_-Ni_3_S_2_ which confirms that their Ni had a lower valence state than in Ni_3_S_2_ because of the sulfur defects, which is consistent with the XPS results [29]. Figure S25 shows the Ni K-edge k3χ(k) oscillatory functions of MI-Ni_3_S_2_, H_2_-Ni_3_S_2_, and Ni_3_S_2_, and their Fourier transformed (FT) curves and the least-square fittings are shown in Fig. 3b and Table S4, and the Ni-S coordinations and the obtained bond lengths are listed in the Table it includes. The Ni-S bond length decreases in the order Ni_3_S_2_ (2.241 Å) > MI-Ni_3_S_2_ (2.238 Å) > H_2_-Ni_3_S_2_ (2.225 Å). The formation of defects leads to lattice collapse and shorter Ni-S bond lengths, but too short a bond length is not conducive to generating unsaturated active sites [43]. Besides, H_2_-Ni_3_S_2_ with many surface sulfur defects has more severe distortion, leading to a shorter Ni-S bond length than in MI-Ni_3_S_2_, possibly making it more unstable.

To seek for the reason for the high catalytic activity of MI-Ni_3_S_2_, density functional theory (DFT) calculations about adsorption configuration, electronic structure, and the dissociation mechanism of Li_2_S were performed. Based on HRTEM images and EXAFS analysis, we constructed a Ni_3_S_2_ (110) slab model. To reflect the difference in structures, MI-Ni_3_S_2_ and H_2_-Ni_3_S_2_ were respectively represented by structures with only bulk and surface sulfur atoms removed (Fig. S26). Li_2_S adsorbed on the different catalysts was first simulated to analyze the activation of the Li-S bond from a geometrical perspective. As shown in Fig. 3c, the average Li-S bond lengths in the ground state and absorbed on the pristine Ni_3_S_2_ and H_2_-Ni_3_S_2_ surfaces are, respectively, 2.08 Å, 2.41 Å, and 2.45 Å, and increases to 2.50 Å on MI-Ni_3_S_2_, indicating the Li-S bond is liable to rupture [44]. In addition, Li_2_S on MI-Ni_3_S_2_ has the highest system energy of –7.15 eV, compared to the ground state of Li_2_S (–7.98 eV), the Li_2_S adsorbed on Ni_3_S_2_ (–7.37 eV) and H_2_-Ni_3_S_2_ (–7.32 eV), also suggesting that Li_2_S is more easily activated for the MI-Ni_3_S_2_ case.

Analyzing optimized adsorption configurations helps clarify the positions of active sites in the different systems, and to reveal the mechanism by which MI-MS selectively binds Li_2_S. As shown in Fig. 3d–f and Fig. S27, all Li atoms are adsorbed on the hollow site composed of multiple sulfur atoms. The two active sulfur sites adsorbing the S atoms of Li_2_S are labeled S1 and S2 and have an almost equal charge (S1: 6.21 e *vs.* S2: 6.22 e) in pristine Ni_3_S_2_. In MI-Ni_3_S_2_, S2 (6.23 e) has a higher charge density than S1 (6.18 e), which is different from H_2_-Ni_3_S_2_ (S1: 6.24 e *vs.* S2: 6.20 e). Compared to Ni_3_S_2_ and H_2_-Ni_3_S_2_, the surface sulfur active sites in MI-Ni_3_S_2_ are transferred to those with more charge, providing more sites for Li atom adsorption. Therefore, the Li-S bond in Li_2_S adsorbed on MI-Ni_3_S_2_ is lengthened and tends to be broken to catalyze the Li_2_S decomposition. In addition, the charge density difference of Ni_3_S_2_-Li_2_S (Fig. 3g) illustrates the clear charge transfer from the catalyst surface to Li_2_S, so that the S atom in Li_2_S is more inclined to attach to the active site with more charge. Therefore, the difference in charge distribution induces the position of active site adsorbing S atoms in different systems. This is also why the sulfur atom (S3), which is closer (2.92 Å) to the Li atom, becomes the new active site for Li_2_S adsorption in MI-Ni_3_S_2_ (Table S5).

The Li_2_S decomposition energies, based on the delithiation process ${\mathrm{L}}{{{\mathrm{i}}}_2}{\mathrm{S\ }} \to {\mathrm{\ LiS}} + {\mathrm{\ L}}{{{\mathrm{i}}}^ + } + {\mathrm{\ }}{{{\mathrm{e}}}^ - }$ (Fig. S28), were also calculated, and show that the energy barriers of Li_2_S decomposition on Ni_3_S_2_, H_2_-Ni_3_S_2_, and MI-Ni_3_S_2_ are 1.08, 0.51, and 0.17 eV, respectively (Fig. 3h), which is consistent with the experimental results. On this basis, we established a correlation between the Li-S bond length and the charge distribution gap of active S atoms (S1 and S2) in Fig. 3i. The insights from the DFT calculations indicate that the selective binding defects in MI-Ni_3_S_2_ with more uneven charge distribution on surface S active sites will facilitate the migration of Li_2_S adsorption sites, ultimately resulting in elongation of the Li-S bond, thereby reducing the transition state energy during its decomposition and increasing the conversion kinetics of Li_2_S.

### Effect of MI catalysts on SRR and SER

**Figure 4.**
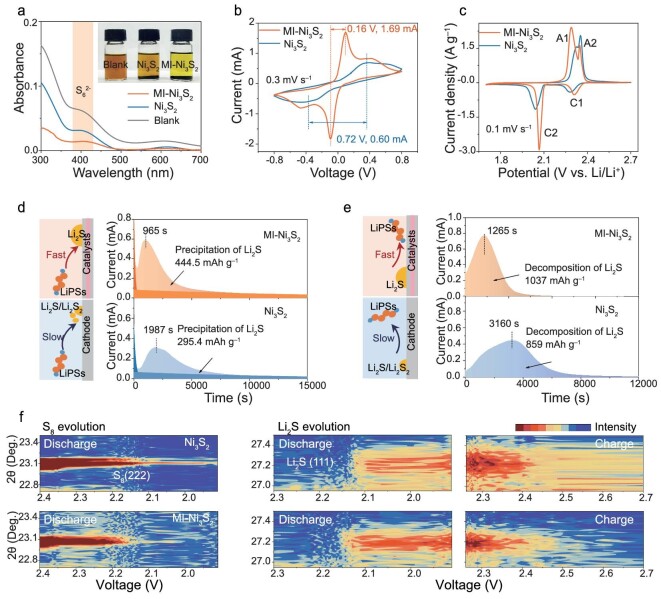
Kinetic performance of the MI-Ni_3_S_2_ catalyst. (a) The UV-visible absorption spectra of Li_2_S_6_ solution with Ni_3_S_2_ and MI-Ni_3_S_2_, and a blank sample (initial Li_2_S_6_ solution without any catalyst). The inset is a photo of the samples. (b) CV curves of symmetric cells with MI-Ni_3_S_2_ and Ni_3_S_2_. (c) The second cycle of CV profiles for cells with MI-Ni_3_S_2_ and Ni_3_S_2_ at a scan rate of 0.1 mV s^−1^. (d and e) Li_2_S deposition and dissolution measurements on different surfaces with diagrams of the conversion mechanisms. (d) Potentiostatic discharge profiles of a Li_2_S_8_ solution discharged at 2.05 V on different surfaces. The dark orange indicates the reduction of Li_2_S_8_/Li_2_S_6_, and the light orange shows the precipitation of Li_2_S. (e) Potentiostatic charge profiles at 2.35 V on different electrodes to evaluate the dissolution behavior of Li_2_S. (f) *In-situ* XRD patterns of S_8_ (222) and Li_2_S (111) evolution in a sulfur cathode during discharge and charge with Ni_3_S_2_ and MI-Ni_3_S_2_ catalysts.

MSs have been widely reported for use in Li-S batteries as catalysts for the SRR due to their good adsorption ability for LiPSs and catalytic activity [45,46]. MI-Ni_3_S_2_ also has a strong adsorption ability for LiPSs, as suggested by a static adsorption test in a 0.01 M Li_2_S_6_/(DME/DOL = 1:1) solution (Fig. 4a and Fig. S29) [33]. Symmetric cells with different catalysts were also studied to show their activities in the redox process [12]. As shown in Fig. 4b and Fig. S30, a cell with MI-Ni_3_S_2_ has the smallest peak separation and the largest redox peak current (0.16 V, 1.69 mA), representing the fastest conversion rate of Li_2_S_6_ to S_8_/Li_2_S, compared to cells with Ni_3_S_2_ (0.72 V, 0.60 mA), and H_2_-Ni_3_S_2_ (0.42 V, 1.04 mA) [47]. The CV profiles with normalized sulfur mass in Fig. 4c and Fig. S31 have two pairs of redox peaks for Li-S cells assembled with a CNT/S cathode. For the reduction process, the two cathodic (reduction) peaks (C1, C2) correspond to the reduction of S_8_ to LiPSs and then to Li_2_S. Compared to one with Ni_3_S_2_, a cell with MI-Ni_3_S_2_ has a positive peak shift and a higher current for the reduction peaks [48]. For the oxidation process, the conversion from Li_2_S to LiPSs and then to elemental sulfur has two anodic peaks ∼2.30 V (A1) and 2.36 V (A2). Compared to a cell with Ni_3_S_2_, one with MI-Ni_3_S_2_ has a much stronger A1 peak that shifts from 2.33 V to 2.28 V. The larger overpotential causes the two oxidation peaks for a cell with Ni_3_S_2_ to merge [47]. In addition, an overlapping CV profile for the first three cycles was observed for the cell with MI-Ni_3_S_2_, but the peak intensity decreased for the one with Ni_3_S_2_ due to the inevitable sulfur loss (Fig. S32). These results suggest that MI-Ni_3_S_2_ has good activity to improve both the SRR and SER.

We also investigated the deposition and dissolution of Li_2_S on different catalysts (Fig. 4d and Fig. S33a). Li_2_S deposition on MI-Ni_3_S_2_ generates an earlier current peak (965 s) and a higher discharge capacity (444.5 mAh g^–1^) than those of H_2_-Ni_3_S_2_ (1057 s, 348.1 mAh g^–1^) and Ni_3_S_2_ (1987 s, 295.4 mAh g^–1^), indicating much better reduction kinetics from LiPSs to Li_2_S. Figure S34 shows the uniform and dense Li_2_S deposition on MI-Ni_3_S_2_, whose selective binding defects generate abundant active sites to improve the deposition of Li_2_S by facilitating three-dimensional spatial contact with the conducting agent and the LiPSs. For the oxidation of the deposited Li_2_S (Fig. 4e and Fig. S33b), the MI-Ni_3_S_2_ electrode also has an earlier current peak (1265 s) and a higher oxidation capacity (1037 mAh g^−1^) than H_2_-Ni_3_S_2_ (1678 s, 910 mAh g^−1^) and Ni_3_S_2_ (3160 s, 859 mAh g^−1^) electrodes, suggesting the fast oxidation of Li_2_S on MI-Ni_3_S_2_, in agreement with the above discussion. SEM images after the oxidation test still show residual Li_2_S on the Ni_3_S_2_ surface (Fig. S35a), in contrast to the clean surface of MI-Ni_3_S_2_ (Fig. S35b). These results support the better bidirectional catalytic activity of MI-Ni_3_S_2_, especially for its significantly better activity for the SER.

Figure 4f shows the *in-situ* XRD analysis of overall sulfur conversion in batteries with the assistance of Ni_3_S_2_ with/without the MI treatment. The S_8_ (222) peak gradually disappears during discharge with the MI-Ni_3_S_2_ catalyst, but the sulfur signal can still be detected for the battery with Ni_3_S_2_ throughout the whole cycling. At the same time, the Li_2_S (111) peak appears much earlier with MI-Ni_3_S_2_ catalyst, suggesting it promotes the LiPSs-to-Li_2_S conversion in the discharge process. Moreover, during charging, the disappearance of Li_2_S is much faster with MI-Ni_3_S_2_ and occurs at a lower potential than with Ni_3_S_2_, proving the excellent activity of MI-Ni_3_S_2_ in the SER. In addition, the characteristic peak for the (110) plane of Ni_3_S_2_ remains stable in both catalysts during cycling (Fig. S36), suggesting its good structural stability in the battery reactions. *In-situ* EIS tests were conducted between 1.8 and 2.7 V for the first two cycles and fitted by a simplified-contact Randles-equivalent circuit. According to the fitted results in Figs S37 and S38, the *R*_ct_ of MI-Ni_3_S_2_ is significantly lower than that of Ni_3_S_2_ in the oxidation process in the first cycle, suggesting improved Li_2_S conversion kinetics and complete conversion. Subsequently, in the reduction process of the second cycle, the *R*_ct_ of the battery with MI-Ni_3_S_2_ is reduced, whereas that of the battery with Ni_3_S_2_ increases significantly with the accumulation of dead Li_2_S due to incomplete oxidation. These results prove that the complete oxidation of Li_2_S facilitates subsequent battery cycling.

### Stability of MI catalysts upon cycling

**Figure 5.**
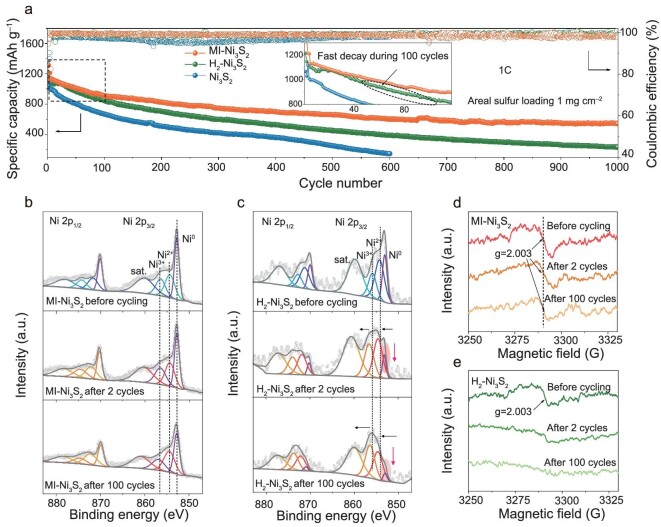
Catalyst stability in battery cycling. (a) Cycling performance of batteries with areal sulfur loading of 1 mg cm^−2^. The inset magnifies the first 100 cycles to show the capacity decay trends. (b and c) Ni 2p XPS spectra. (d and e) EPR spectra of cathodes with MI-Ni_3_S_2_ and H_2_-Ni_3_S_2_ after different numbers of cycles.

A Li-S battery with MI-Ni_3_S_2_ as the catalyst has a high initial capacity of 1388 mAh g^−1^ at 0.1C (sulfur utilization ∼85%) and maintains excellent cycling stability with a low capacity-decay rate of 0.05% over 1000 cycles at 1C (Fig. 5a). Batteries with the MI-Ni_3_S_2_ and H_2_-Ni_3_S_2_ catalysts have almost the same initial specific capacities (1131 and 1129 mAh g^−1^, respectively) at 1C, and the same capacity retention rate for about the first 40 cycles. However, H_2_-Ni_3_S_2_ then experiences a rapid capacity decay, which becomes more severe after 100 cycles (Fig. S39). The stability of these two catalysts in Li-S batteries was therefore examined [48]. As shown in Fig. 5b, the Ni 2p XPS spectra of MI-Ni_3_S_2_ show no shift for Ni^0^, Ni^2+^, and Ni^3+^ peaks. The relative peak intensities of Ni^0^/Ni^2+^ species remain unchanged after 2 cycles (1.241 *vs.* 1.236) and then drop slightly to 1.189 after 100 cycles. The EPR results obtained from the electrodes also show no significant changes before and after cycling (Fig. 5c). Moreover, HRTEM imaging shows that the (110) spacing of MI-Ni_3_S_2_ in the cathode after cycling is 2.73 Å (Fig. S40), which is similar to the initial MI-Ni_3_S_2_ (2.72 Å in Fig. 1e). These results suggest the valence state of the Ni species remains stable and the MI sites are still present after cycling. In sharp contrast, H_2_-Ni_3_S_2_ shows an apparent shift of the Ni^2+^ and Ni^3+^ peaks to higher binding energies after cycling (Fig. 5b), and the peak area of Ni^0^ decreases significantly. The EPR spectra also show that the defect signal gradually disappeared in subsequent cycles (Fig. 5d). These results indicate much better stability of bulk MI sites in MI-Ni_3_S_2_ than the usual surface sulfur vacancies in long-term cycling.

**Figure 6.**
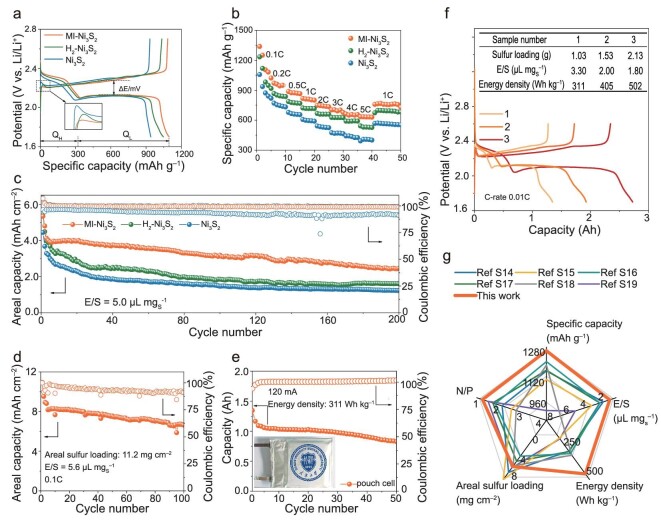
Performance of Li-S batteries with the MI-Ni_3_S_2_ catalyst. (a) Galvanostatic discharge-charge profiles at 0.2C with various catalysts. The inset is the enlarged charging potential barrier. (b) Rate performance. (c) Cycling performance of batteries at E/S = 5.0 μL mg_S_^−1^ of sulfur cathodes with various catalysts. (d and e) Cycling performance of the batteries with (d) high-sulfur loading at 0.1C, and (e) pouch cell at 0.07C, with MI-Ni_3_S_2_. (f) Galvanostatic discharge-charge profiles at 0.01C of three MI-Ni_3_S_2_-based pouch cells with different energy densities. The inset table gives the parameters of the batteries. (g) Performance comparison between this work and previously reported Li-S pouch cells. N/P, negative/positive ratio signifying ratio between anode and cathode capacity.

An enlarged view of the charging curve in Fig. 6a shows that the battery with MI-Ni_3_S_2_ has a much lower potential barrier than others during charging, indicating a significantly increased conversion from Li_2_S to LiPSs. During discharge, the low voltage plateau (Q_L_) of the battery with MI-Ni_3_S_2_ is also much longer, indicating improved kinetics of the LiPS-to-Li_2_S conversion, in agreement with the results of the previous experiment. Thus, the battery with MI-Ni_3_S_2_ has the best rate capability and achieves high reversibility of 655 mAh g^−1^ at 5C (Fig. 6b) with two well-defined voltage plateaus and low polarization due to the improved reaction kinetics and decreased dead Li_2_S formation, in contrast to the high polarization and much-shorter plateaus for both batteries with H_2_-Ni_3_S_2_ and Ni_3_S_2_ (Fig. S41). As shown in Fig. S42, MI-Ni_3_S_2_ has the smallest polarization voltage gap (160 mV) between anodic and cathodic sweeps among the different samples at a C-rate of 0.2C, which increases to 210 mV at 5C. This value is considerably lower than those of H_2_-Ni_3_S_2_ (331 mV) and Ni_3_S_2_ (407 mV). The Li^+^ diffusion coefficient (*D_Li^+^_*) was calculated using the Randles-Sevcik equation based on the CV profiles at different scanning rates, and showed the highest Li ion diffusion ability of the battery with MI-Ni_3_S_2_ compared with those with H_2_-Ni_3_S_2_ and Ni_3_S_2_ (Figs S43 and S44), due to the greatly suppressed dead Li_2_S formation in the electrodes [49]. The cycling performance using a lean electrolyte was also investigated for cells with different catalysts. With a high sulfur areal loading of up to 5.0 mg cm^–2^ and low E/S ratios (8.5 μL mg_S_^−1^ and 6.4 μL mg_S_^−1^), the batteries with MI-Ni_3_S_2_ have high initial areal capacities of up to 7.57 mAh cm^−2^ and 6.16 mAh cm^−2^, respectively, at 0.05C, and also shows good cycling stability for >200 cycles (Fig. S45). Even when E/S is as low as 5.0 μL mg_S_^−1^, the battery with MI-Ni_3_S_2_ has a much lower overpotential and higher areal capacity at 0.1C than H_2_-Ni_3_S_2_, and Ni_3_S_2_, indicating that MI-Ni_3_S_2_ with selective binding defects obviously improves the conversion kinetics under lean electrolyte conditions (Fig. S46). Besides, as shown in Fig. 6c, the MI-Ni_3_S_2_ battery with a high initial areal capacity of 5.34 mAh cm^−2^ still has an areal capacity of 2.46 mAh cm^−2^ after 200 cycles, much higher than the equivalent values for H_2_-Ni_3_S_2_ (1.60 mAh cm^−2^) and Ni_3_S_2_ (1.23 mAh cm^−2^). The electrochemical performance tests indicate that the MI-Ni_3_S_2_ catalyst significantly improved the performance of the Li-S battery and the redox reaction kinetics of sulfur and Li_2_S.

When the areal sulfur loading increases to 11.2 mg cm^–2^ (E/S = 5.6 μL mg_S_^−1^), the battery with MI-Ni_3_S_2_ has a high initial area capacity of over 10.95 mAh cm^–2^, and its reversible capacity maintains at 6.65 mAh cm^–2^ over 100 cycles at 0.1C (Fig. 6d). Moreover, to demonstrate the practical use of Li-S batteries, we assembled an Ah-level pouch cell with MI-Ni_3_S_2_ which had an initial capacity of 1.35 Ah under a current density of 117 mA g_S_^–1^, and a specific energy density of 311 Wh kg^−1^, with stable cycling over 50 cycles (Fig. 6e). Other Ah-level cells employing the MI-Ni_3_S_2_ as the catalyst deliver even the superhigh initial of 405 Wh kg^−1^ and 502 Wh kg^−1^ (Fig. 6f) with a low E/S (2.0 μL mg_S_^−1^, 1.8 μL mg_S_^−1^, respectively, details are presented Table S6 and Fig. S47). Fig. 6g and Fig. S48, which shows the key parameters of our pouch cell with the MI-Ni_3_S_2_ catalyst compared to the reported practical Li-S batteries [50], show that our cell is superior.

## CONCLUSION

We propose an electrochemically molecular-imprinting strategy to synthesize selective binding defects in MS catalysts for Li_2_S electrocatalysis in Li-S batteries, a rate-limiting step challenging battery reversibility. By removing the imprinted molecular Li_2_S in MSs, vacancies are created in the bulk, which induces a charge redistribution of the surface sulfur atoms. This strengthens the binding with Li atoms and consequently stretches the Li-S bond in Li_2_S reactants, forming a molecular-bound, smooth solid-solid interface to reduce its oxidation barrier. The MI-Ni_3_S_2_ has the best catalytic activity for Li_2_S oxidation among the MI-MS catalysts examined due to the highest vacancy content. It shows the bidirectional catalytic activity both for SRR and SER, leading to a low apparent *E*_a_ in the discharge-charge process (0.25–0.35 eV). Due to the accelerated sulfur reaction, an Ah-level pouch cell with 311 Wh kg^−1^ shows excellent cycling performance, while a cell featuring an energy density up to 502 Wh kg^−1^ with an ultralow E/S ratio of 1.8 μL mg_S_^−1^ demonstrates its potential in lean-electrolyte operation. Our work presents a fundamental solution to the problem of catalysis governed by suboptimal solid-solid interface coupling not limited to sulfur-based batteries discussed here. The electrochemically molecular-imprinting strategy also possesses versatility for general MI catalyst syntheses by selecting a specific reaction.

## MATERIALS AND METHODS

### Materials synthesis

The necessary process for producing MS-MI is lithiation followed by delithiation. First, 80 wt% of the metal sulfide powder (FeS_2_, CoS_2_, CuS, Ni_3_S_2_, MnS_2_, WS_2_, and MoS_2_) was mixed with 10 wt% of multi-wall carbon nanotubes (MWCNTs, Guangdong Canrd New Energy Technology Co., Ltd.) and 10 wt% of polyvinylidene difluoride (PVDF) binder dissolved in N-methyl-2-pyrrolidone (NMP, 98%, Aladdin). It was then coated uniformly on the surface of a fresh aluminum foil and dried at 100°C for ∼8 h to remove the solvent. Next, a lithium-ion cell, using MS/MWCNTs as the cathode with sulfur-loading controlled at 1 mg cm^–2^, a lithium foil as the anode, and 1 M bis-trifluoromethane sulphonyl imide (LiTFSI) in 1, 3-dioxolane and 1, 2-dimethoxyethane (DOL:DME, 1:1 vol) purchased from DoDoChem as the electrolyte, was assembled in the 2032 coin cell under pure argon atmosphere (O_2_ <1 ppm and H_2_O <1 ppm). A current density of 0.10 mA cm^−2^ was applied in the 1–3 V voltage window *vs.* Li^+^/Li. After one cycle of charge and discharge, the MS cathodes were disassembled and rinsed with ethanol several times to remove the electrolyte and Li_2_S by-product (MI-Ni_3_S_2_ (2–10) were carried by 2–10 cycling in batteries). In addition, the commercial Ni_3_S_2_ powder was heated at 500°C for 2 h at a heating rate of 5°C min^−1^ in an H_2_ (5%)/Ar atmosphere to attain H_2_-Ni_3_S_2_.

### Assembly of Li-S pouch cells

The sulfur cathodes used for pouch cells, S/CNT/composites (64 wt% sulfur content), conductive carbon (26 wt%, CNTs), LA133 binder (5 wt%), and MI-Ni_3_S_2_ catalyst (5 wt%) in solid content of 1 g 5 mL^–1^ (H_2_O) were well ground and subsequently dispersed. The obtained slurry was coated on both sides of a carbon-coated aluminum foil with a thickness of 800 μm each side, which was then transferred to a 35°C heated plate to evaporate the H_2_O for >6 h and then dried at 60°C for >12 h, resulting in a sulfur cathode for use in the pouch-cell assembly. The total sulfur loading and areal-sulfur loading of the as-prepared cathodes could be adjusted by changing the amount of S/CNT composite used and the area of the rectangular aluminum foil electrode. This sulfur electrode with the catalyst was used as the cathode, lithium foil (100 μm) was used as the anode, an alumina film was used as the separator, and an Al-plastic film was used as packaging material. The electrolyte was DOL/DME (1:1, by volume) with 0.1 M LiTFSI and 5.0 wt% LiNO_3_ additives. The assembly process was a multi-layer superposition, and both sides of the cell were lithium foil because of the double-coating cathode. Reserve an airbag when the top side of the battery is sealed.

## Supplementary Material

nwae190_Supplemental_File
