# Supplementary material for: Engineering catalytic defects via molecular imprinting for high energy Li-S pouch cells
Source: Natl Sci Rev. 2024 May 31;11(7):nwae190. doi: 10.1093/nsr/nwae190 (PMC11210504; doi:10.1093/nsr/nwae190)
Supplement: nwae190_Supplemental_File [file nwae190_supplemental_file.pdf]

Engineering Catalytic Defects via Molecular Imprinting for High Energy Li-S Pouch Cells

Yufei Zhao[1,2,3,†], Chuannan Geng[1,2,†], Li Wang[2,3,†], Yun Cao[1], Haotian Yang[1,2,3], Linkai Peng[1], Xin Jiang[2], Yong Guo[2], Xiaolin Ye[2], Wei Lv[1,*] and Quan-Hong Yang[2,3,*]

[1]Shenzhen Geim Graphene Center, Engineering Laboratory for Functionalized Carbon Materials, Tsinghua Shenzhen International Graduate School, Tsinghua University, Shenzhen 518055, China;

[2]Nanoyang Group, Tianjin Key Laboratory of Advanced Carbon and Electrochemical Energy Storage, School of Chemical Engineering and Technology, and Collaborative Innovation Center of Chemical Science and Engineering (Tianjin), Tianjin University, Tianjin 300072, China;

[3]Joint School of National University of Singapore and Tianjin University, International Campus of Tianjin University, Fuzhou 350207, China

***Corresponding authors**. E-mails: lv.wei@sz.tsinghua.edu.cn; qhyangcn@tju.edu.cn

†Equally contributed to this work.

Methods

Materials Characterization

The morphology of the Ni_3S_2, MI-Ni_3S_2, and H_2-Ni_3S_2 and the electrodes were characterized by scanning electron microscopy (SEM) on a Regulus 8100 (Hitachi Japan) instrument with an accelerating voltage of 5.0 kV. High-resolution transmission electron microscopy (HRTEM) observations were made on a JEM 2100F coupled with an energy dispersive spectrometer (EDS), aberration corrected transmission electron microscope (Thermo Fisher Scientific, Spectra 300) operating at 200 kV contributed to Spherical aberration-corrected high-angle annular dark-field scanning transmission electron microscope (HAADF-STEM) images. The Raman spectra were recorded on a Raman system (Horiba Lab RAM HR800) with an argon ion laser (532 nm). XRD patterns were collected on a Bruker D-8 diffractometer (Cu Kα radiation, λ = 0.154 nm) at room temperature; XPS measurements were made using a Thermo Fisher Scientific K-Alpha+ unit with Al Kα radiation, hv = 1486.6 eV. UV-visible adsorption tests were carried out using a Thermo UV-visible spectrophotometer. EPR measurements were made using a JES-FA200 (Japan) apparatus. X-ray absorption spectroscopy (XAS) measurements were performed at the 4B9A beamline of BSRF under ring conditions of 2.2 GeV and about 80 mA. A Si (111) double-crystal monochromator was used for energy selection, and the sample data were collected in the fluorescence mode, while data on the standards (Ni foil, NiO) were collected in the transmission mode. The spectrometer energy resolution was approximately 1.4 eV, which gave an overall energy resolution of about 1.5 eV at the Ni K-edge, including core-hole effects. Energy calibration was performed by simultaneous measurement of the absorption edge of a Ni foil, i.e., referencing the energy shift between the maximum of the first peak in the derivative spectrum of a pure Ni foil and the Ni K-edge reference energy. Data was processed

using the Athena and Artemis software from the IFEFFIT package. cold-field-emission spherical.

Cell assembly and the corresponding electrochemical measurements

The MI-Ni_3S_2-S cathodes were prepared by adding MI-Ni_3S_2 (Ni_3S_2, H_2-Ni_3S_2)-MWCNT powder (10%), sulfur-MWCNT powder (80 wt%) (typically, a mixture of MWCNTs and sulfur with the mass ratio of 2:8 was ground uniformly and heated at 155 °C for 12 h[1]) and PVDF (10 wt%) binder in NMP to form a slurry, which was then coated onto an Al foil and dried at 55 °C for 12 h. A catalyst-free cell was assembled using the same procedure without adding Ni_3S_2 or MI-Ni_3S_2 powders to the cathode slurry. The electrochemical performance was evaluated using a CR2032 coin cell with MI-Ni_3S_2-S cathodes, a lithium foil, a Celgard 2500 separator, and 1.0 mol L^{-1} LiTFSI in a DOL/DME solution (1:1, v/v) with 1.0 wt% $LiNO_3$ from DoDoChem as the electrolyte (E:S ratio=20:1 μL mg_S^{-1}). The sulfur mass loading of the electrodes was typically about 1.0 mg cm^{-2}. The E/S ratios were about 10 μL mg_S^{-1} for electrodes with sulfur loadings of 11.21 mg cm^{-2}. EIS was measured on an Autolab workstation between 100 kHz and 0.01 Hz. A NEWARE Battery Test System (CT-4008T-5V50mA-164，Shenzhen,China) was used to conduct the discharge/charge measurements with a potential range from 1.7-2.7 V (*vs.* Li/Li^+). The in-situ XRD tests used a Rigaku D/MAX 2500/PC X-Ray Diffractometer, with a 2θ range of 20°-60° and a scan rate of 10° min^{-1}. Cu-Kα (λ=0.154056 nm) radiation was used with an operating voltage of 40 kV. The prepared catalyst-containing (30%) sulfur electrode slurry was cast onto an aluminum foil and then cut into a diameter of 16 mm for the cathode. The electrolyte was the same as in the cell assembly section, with 50 μL on each side. The Li-S cell used glass fiber separators with a lithium foil anode. The cell was galvanostatically charged-discharged at 0.1 C with a potential range of 1.7-2.8 V (*vs.* Li/Li^+) on a LAND testing system.

Kinetic study of LiPS conversion

A Li_2S_n solution was prepared by mixing stoichiometric amounts of S and Li_2S in 1.0 mol L^{-1} LiTFSI in a DOL/DME solution (1:1, v/v) and stirred overnight at 50 °C. CV measurements of LiPS conversion were made on an Autolab workstation at a scan rate of 0.1 mV s^{-1} and with a voltage window between 1.7 V and 2.7 V (*vs*. Li/Li^+). The method to assemble full cells is the same as that for electrochemical measurements.

To fabricate the electrodes for symmetric batteries, Ni_3S_2-MWCNT or MI-Ni_3S_2-MWCNT powder was ultrasonically dispersed in NMP, and the dispersion dropped onto carbon paper (CP), which was cut into 10 mm diameter circles after drying for use with the electrodes (Ni_3S_2/CP, MI-Ni_3S_2/CP). They were assembled in a typical CR2032 with a polypropylene (PP) membrane as the separator and 40 µL of Li_2S_6 electrolyte (20 µL for each side of the separator), and with a Voltage window from −0.8 V to 0.8 V at a scan rate of 0.3 mV s^{-1} with Ivium workstation.

Linear sweep voltammetry (LSV) tests were made using a rotating disk electrode (RDE) connected to an electrochemical workstation and a controlled speed rotator (AFMSRCE, Pine Instruments). 10 µL of 2 mg mL^{-1} catalyst ink (made by sonicating 2 mg of catalyst in 1 mL ethanol and 20 µL 5 wt% Nafion solution) was drop-cast onto a glassy carbon electrode (0.196 cm^{-2}) to form a flat film electrode with a loading of 0.1 mg cm^{-2} for all catalysts. The electrochemical test was performed in a three-electrode open cell using lithium foil as the counter and reference electrodes and the catalyst film as the working electrode. The SER test was performed with a sweep rate of 10 mV s^{-1} in the voltage range of −0.8 V to 1 V (*vs*. Ag/Ag^+), using the catalyst film, 0.1 M Li_2S/methanol, a platinum sheet, and Ag/AgCl electrodes as the working electrode, electrolyte, and counter and reference electrodes, respectively[2, 3].

Li_2S nucleation test

Deposition by potentiostatic (2.02 V) discharge experiments was tested by using an Autolab workstation, using test cells (Ni_3S_2/CP, MI-Ni_3S_2/CP, and H_2-Ni_3S_2/CP as the cathodes and lithium foils as the anodes, and LiPSs (0.2 M Li_2S_8|1.0 M LiTFSI in DOL/DME=1:1) as the catholyte). The assembled batteries were first galvanostatically discharged under a current of 0.1 mA until the voltage was 2.1 V and then discharged potentiostatically at 2.02 V for Li_2S deposition[4].

Li_2S dissolution test

The cells were first galvanostatically discharged to 1.70 V at 0.10 mA, then galvanostatically discharged to 1.80 V at 0.01 mA to ensure the sufficient transformation of LiPSs into solid Li_2S on the catalyst surface. Afterward, the cells were potentiostatically charged at 2.35 V until the charge current was below 10^{-5} A for the complete dissolution of the Li_2S[4].

Li_2S electrode preparation

Li_2S cathodes were prepared by mixing Li_2S-MWCNT powder (75 wt%), MWCNT powder (5 wt%) as a conductive additive, and poly (vinylidene fluoride) (10 wt%) as a binder dissolved in N-methyl-2-pyrrolidone (NMP), and 10 wt% of the different catalysts to form a slurry, which was then coated onto an Al foil and dried at room temperature for 48 h in an Ar atmosphere. The dried foil was then cut into a 10 mm diameter circle for use as the cathode.

Density functional theory (DFT) calculation details

All spin-polarized DFT calculations were based on the Vienna *Ab Initio* Simulation Package (VASP) code[5]. The generalized gradient approximations (GGA) with the Perdew-Burke-Ernzerhof (PBE) function were chosen to describe the exchange and correlation energy[6]. The projector augmented wave (PAW) method was used to treat the interactions between electrons and ions[7]. An

energy cutoff of 400 eV was used based on the plane wave functions. The convergence criterion for energy between two electronic steps was 10^{-6} eV, and structural optimization was performed until the maximum force on each atom was less than 0.01 eV $Å^{-1}$. For Ni_3S_2 bulk calculations (a=5.72 Å, b=5.72 Å, and c=7.14 Å), we used a Γ-centered k-mesh of $(5 \times 5 \times 5)$ for Brillouin zone sampling. In the Ni_3S_2 (110) surface calculations (a=14.27 Å, b=9.91 Å, and c=25.71 Å), a Γ-centered k-mesh of $(1 \times 2 \times 1)$ was used. A vacuum with a thickness of 15 Å was used to avoid periodic slab-slab interactions. During the simulation of LiPS adsorption, the DFT+D_3 method was applied to describe the van der Waals interaction (vdWs) between Li_2S and the catalyst[8]. The Li_2S decomposition barrier was simulated using the climbing image nudged elastic band (CINEB) method.

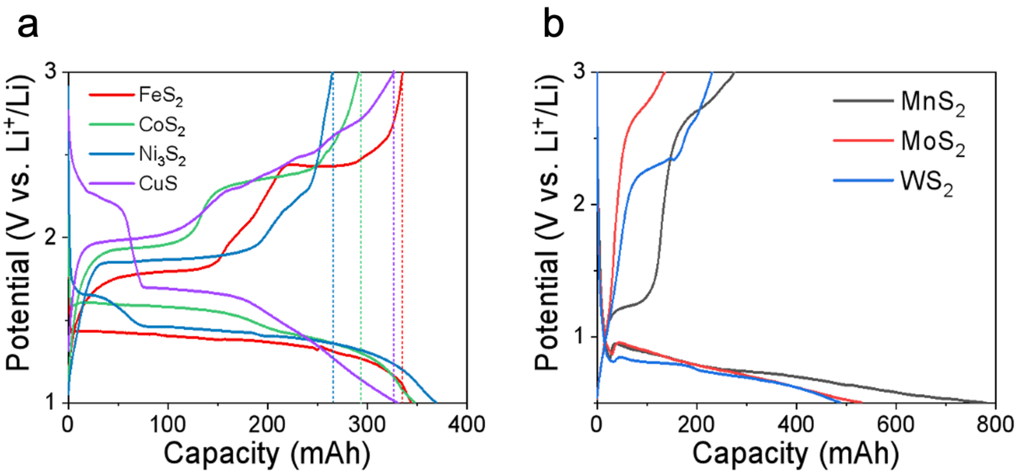

Fig. S1 Discharge and charge curves of various MSs using Li metal as a counter electrode in the first cycle.

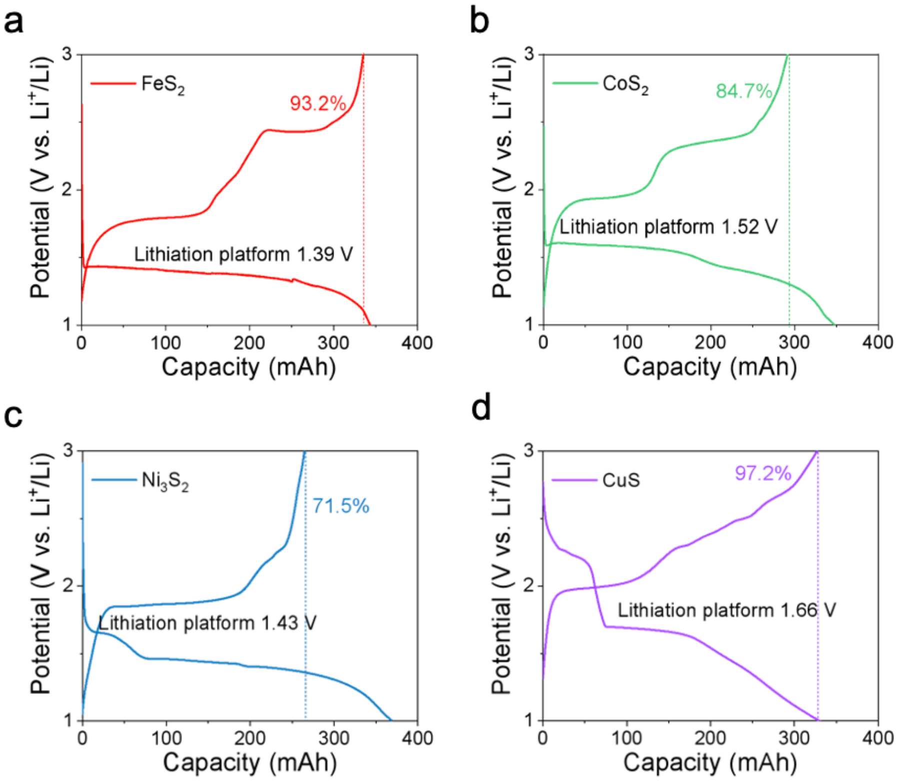

Fig. S2 Lithiation platforms and initial coulombic efficiencies of the half-cells with different cathode revealing by discharge and charge curves. (a) FeS_2, (b) CoS_2, (c) Ni_3S_2, and (d) CuS.

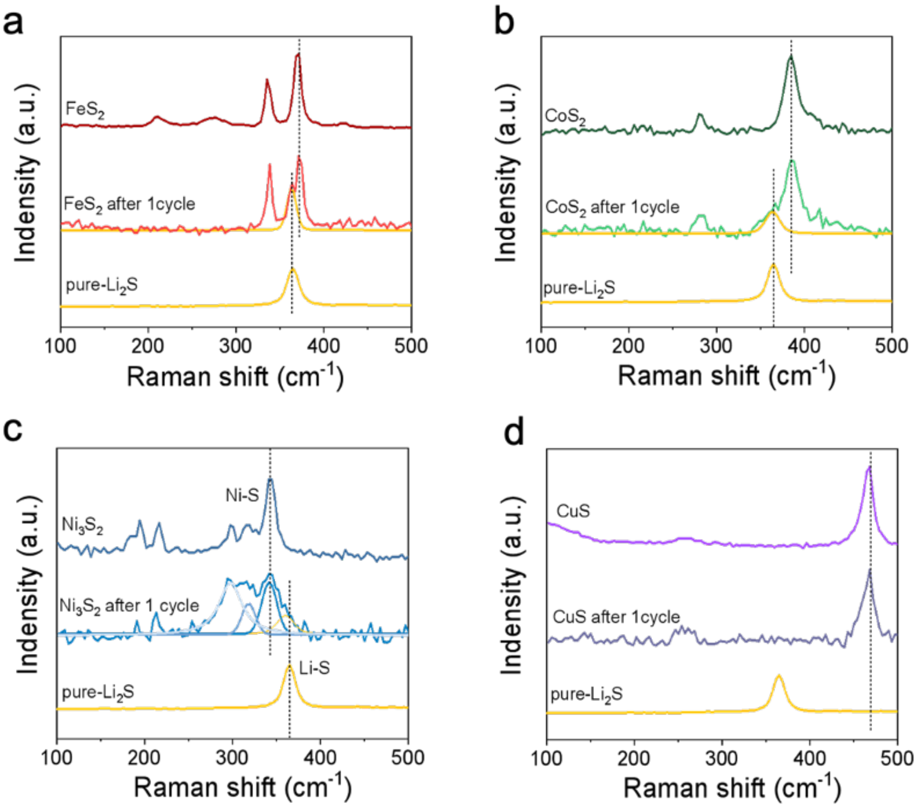

Fig. S3 Raman spectra before and after one cycle in the half-cells. (a) FeS_2, (b) CoS_2, (c) Ni_3S_2, and (d) CuS.

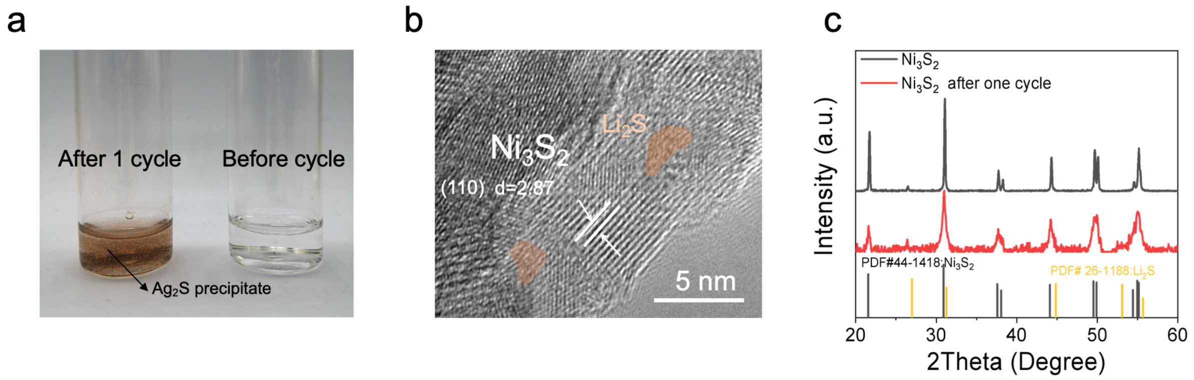

Fig. S4 (a) Titration experiment by $AgNO_3$ with Ni_3S_2 before and after one cycle. (b) HRTEM images of Ni_3S_2. with Li_2S. (c) XRD pattern of Ni_3S_2 after one cycle.

Note: The Ni_3S_2 electrode that was not cleaned with ethanol after the cycle was immersed in water. After the solution was stabilized, the supernatant was taken, and an appropriate amount of $AgNO_3$ solution was used for the titration experiment. As shown in Fig. S4a, the yellowish brown Ag_2S precipitation appeared in the Ni_3S_2 supernatant after one cycle, which was in sharp contrast to the initial clarified soaking solution of Ni_3S_2, strongly indicating the existence of Li_2S in Ni_3S_2 after cycle.

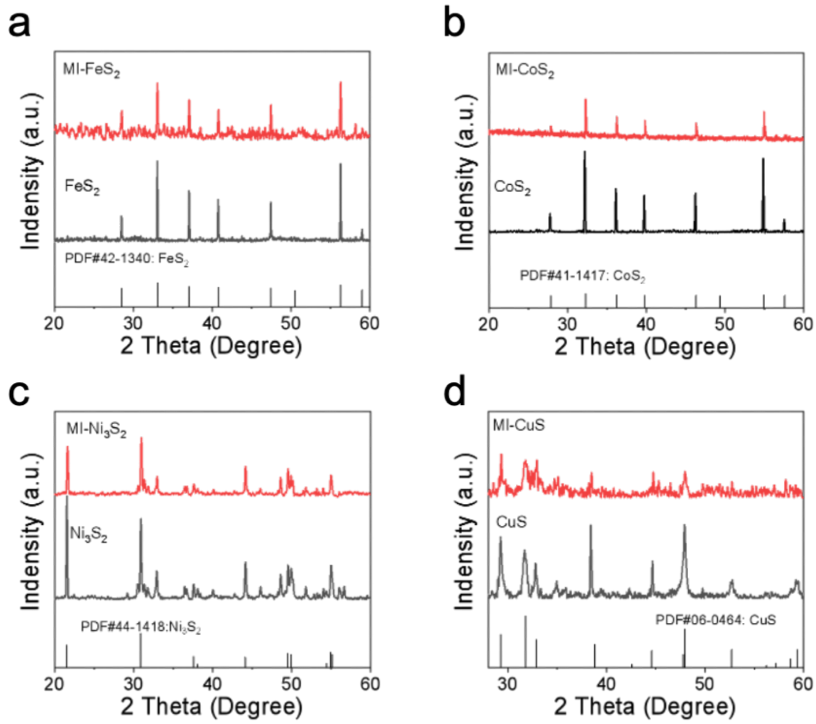

Fig. S5 XRD patterns of MI-MSs and their original counterparts. (a) MI-FeS_2, (b) MI-CoS_2, (c) MI-Ni_3S_2, and (d) MI-CuS

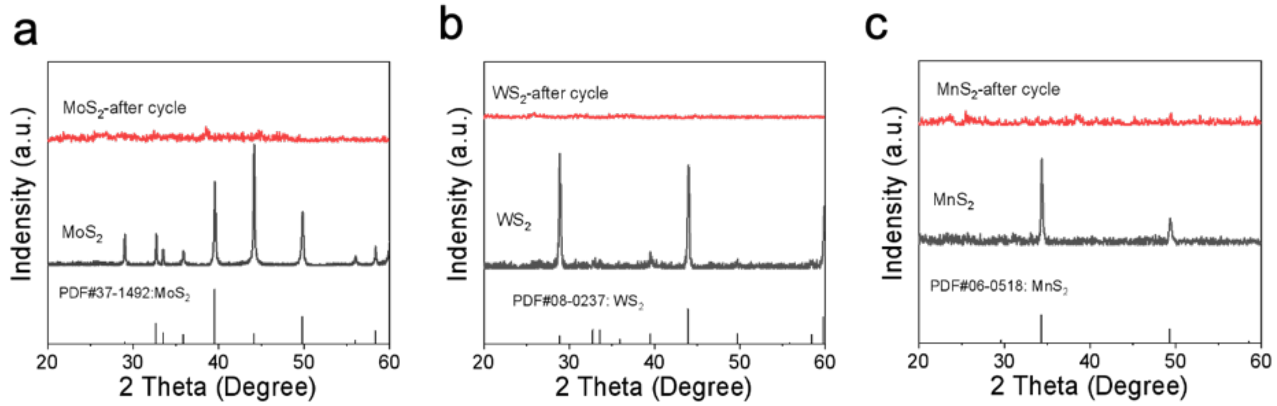

Fig. S6 XRD patterns of layered MSs before and after one cycle in the half-cells. (a) MoS_2, (b) WS_2, and (c) MnS_2.

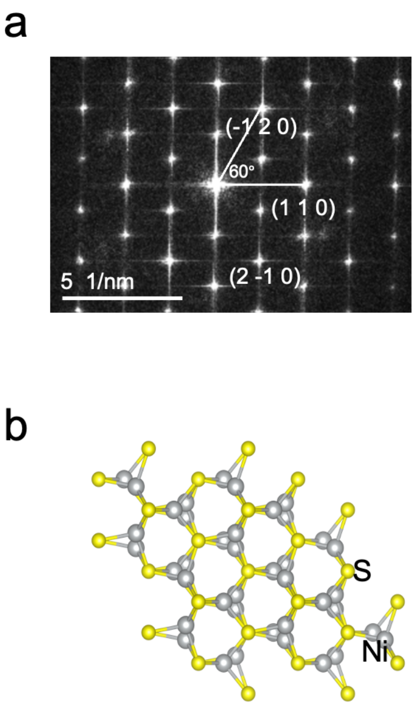

Fig. S7 (a) FFT (HAADF) of the image with the [001] zone axis, and (b) corresponding structure diagram with this axis of Ni_3S_2.

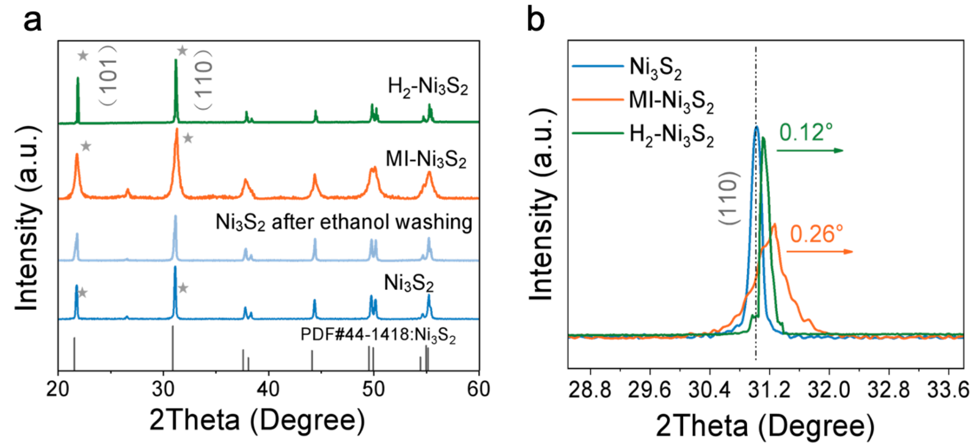

Fig. S8 XRD patterns. (a) Ni_3S_2, Ni_3S_2 after ethanol washing, MI-Ni_3S_2, and H_2-Ni_3S_2, (b) Magnified patterns of their (110) peaks.

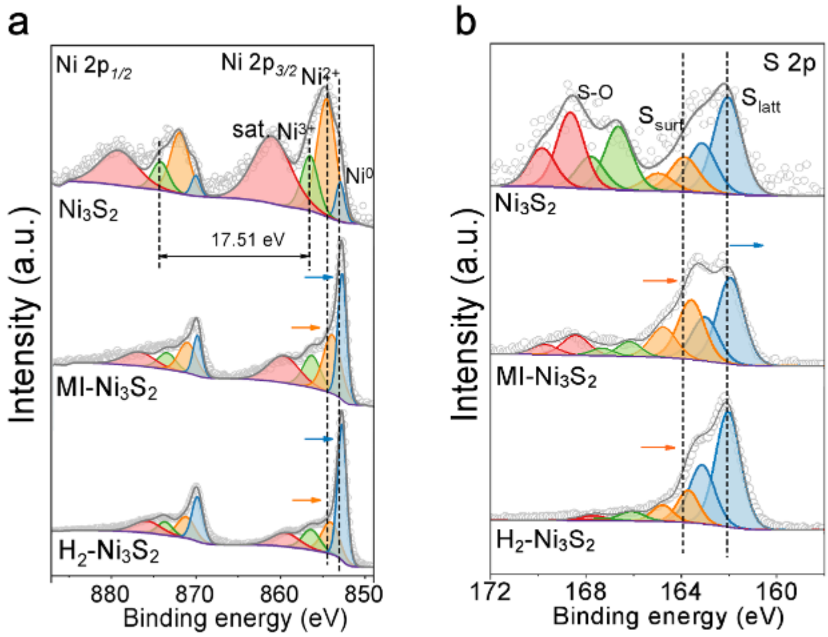

Fig. S9 XPS spectra. (a) Ni 2p and (b) S 2p of MI-Ni_3S_2, H_2-Ni_3S_2, and Ni_3S_2.

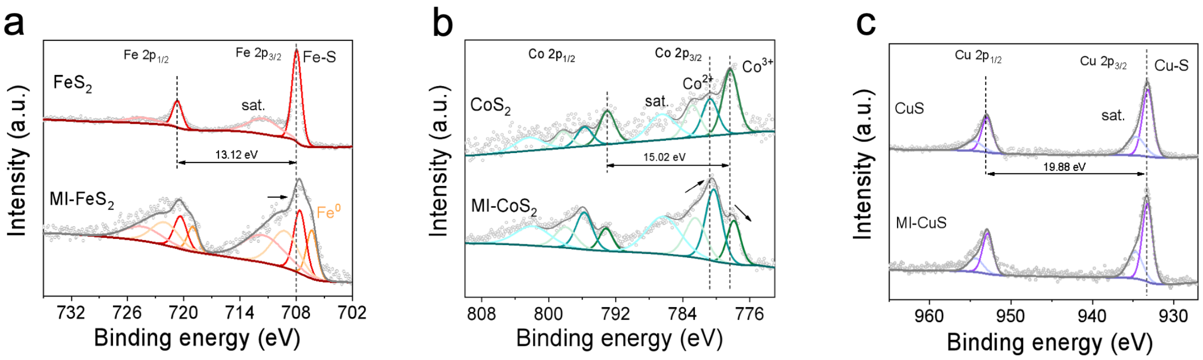

Fig. S10 Fe 2p, Co 2p, and Cu 2p XPS spectra. (a) MI-FeS_2 and FeS_2, (b) MI-CoS_2 and CoS_2, (c) MI-CuS and CuS.

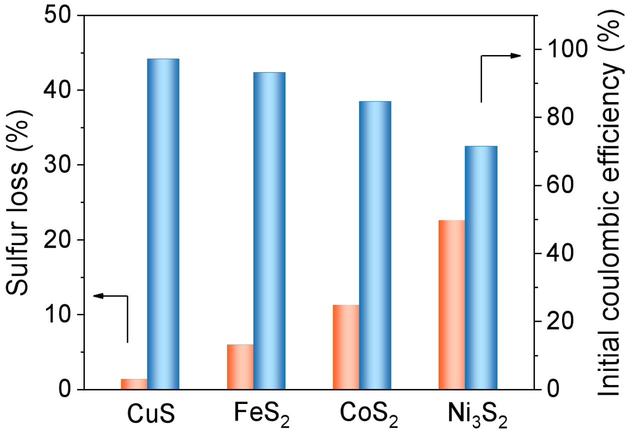

Fig. S11 Sulfur losses of four MI-MSs (ICP-MS results) and their initial coulombic efficiencies in half-cells.

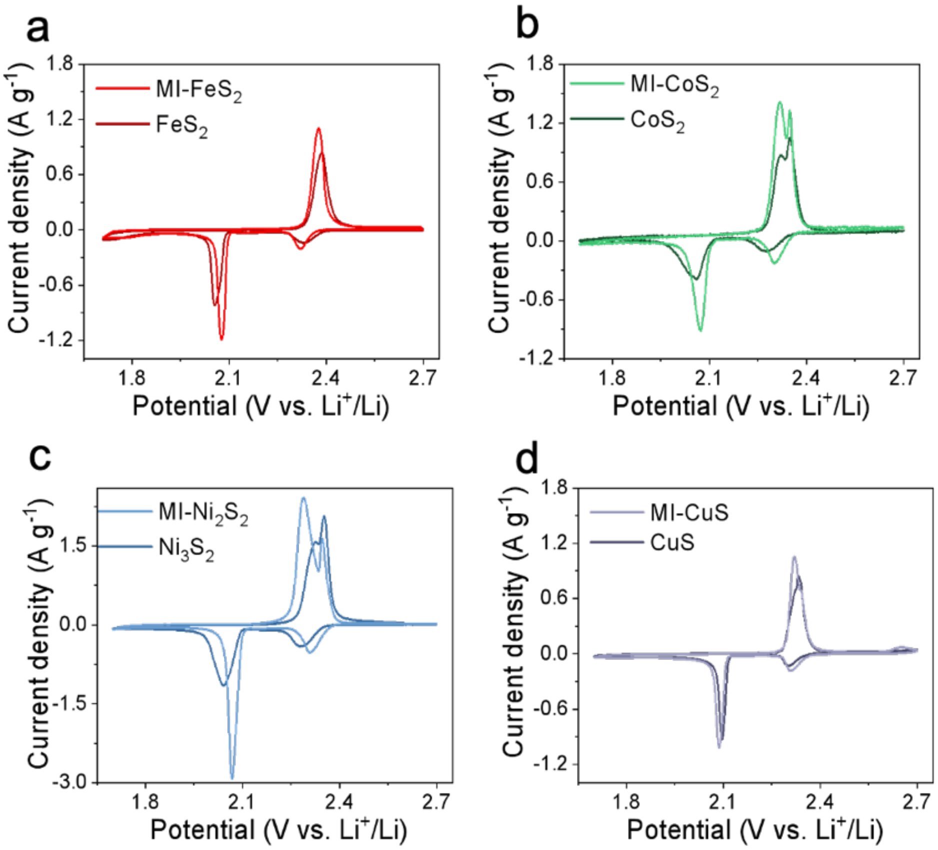

Fig. S12 CV curves of assembled Li-S cells with MI-MS and MS catalysts at a scan rate of 0.1 mV s^{-1}. (a) MI-FeS_2 and FeS_2, (b) MI-CoS_2 and CoS_2, (c) MI-Ni_3S_2 and Ni_3S_2, and (d) MI-CuS and CuS.

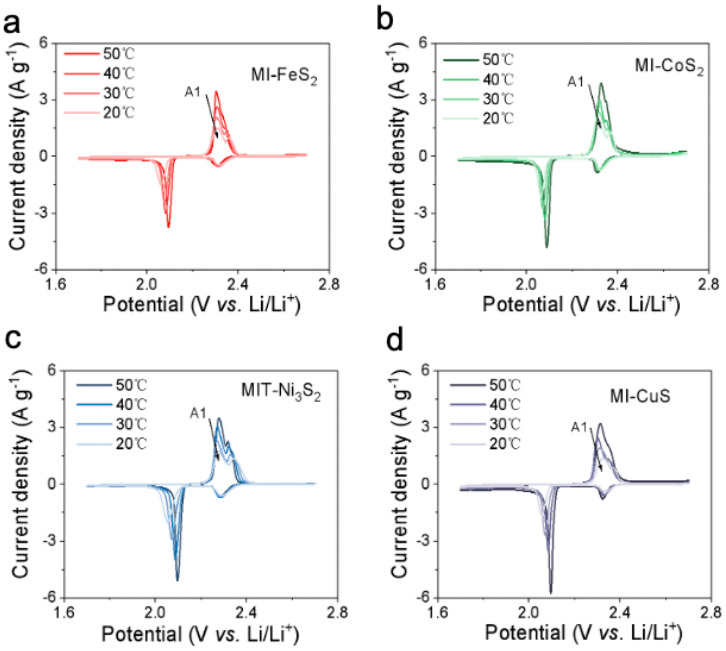

Fig. S13 CV curves of Li-S cells with various catalysts. (a) MI-FeS_2, (b) MI-CoS_2, (c) MI-Ni_3S_2, and (d) MI-CuS at 20 °C, 30 °C, 40 °C, and 50 °C (scan rate: 0.1 mV s^{-1}).

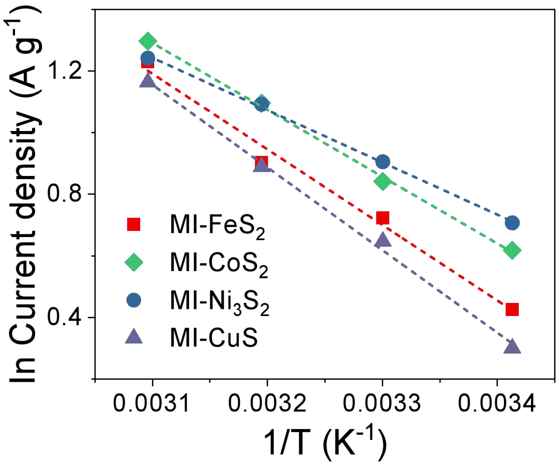

Fig. S14 The relationships between the peak currents of Li_2S oxidation and temperatures for different MI catalysts.

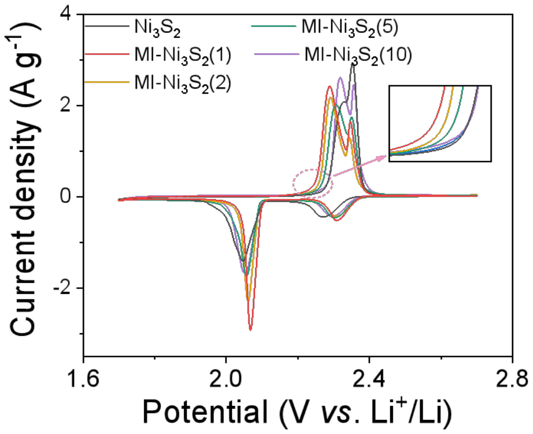

Fig. S15 CV curves of Li-S cells with MI-Ni_3S_2 (1-10) at a scan rate of 0.1 mV s^{-1}.

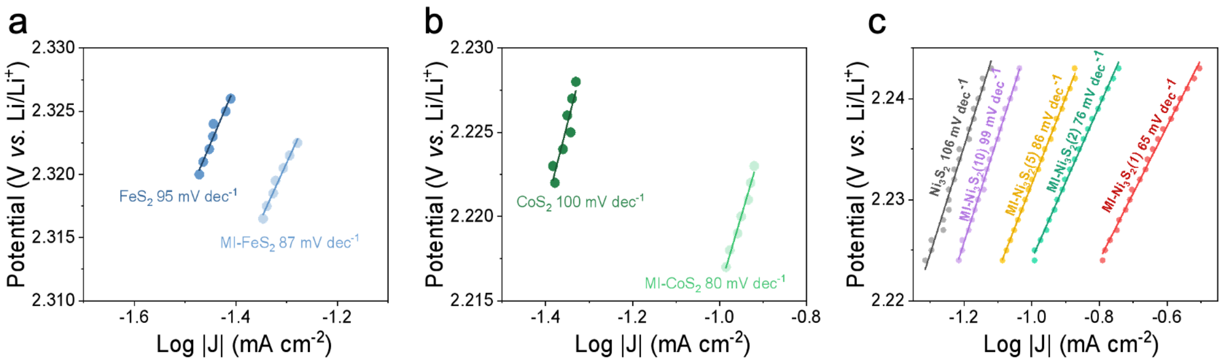

Fig. S16 The fitted Tafel plots corresponding to the oxidation peak of Li_2S-LiPSs in CV curves with various catalysts. (a) FeS_2 and MI-FeS_2, (b) CoS_2 and MI-CoS_2, and (c) Ni_3S_2 and MI-Ni_3S_2 (1-10).

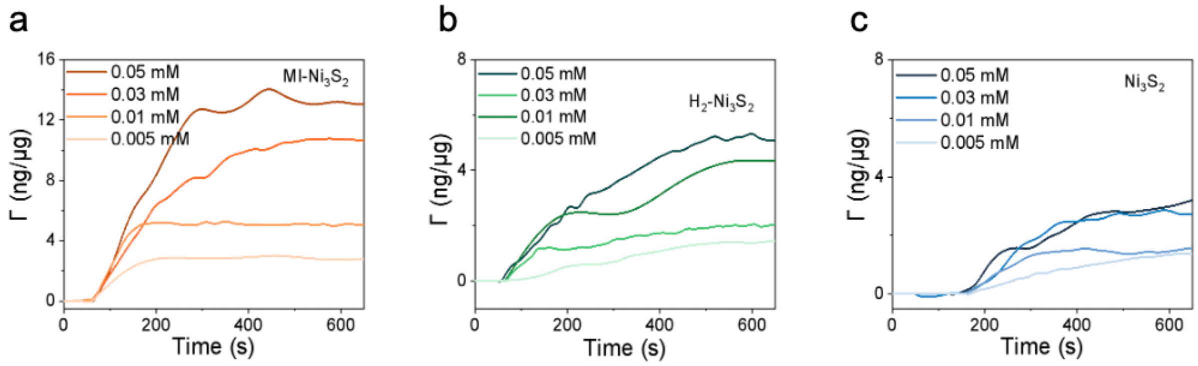

Fig. S17 Mass change curves of various catalysts in Li_2S solutions with different concentrations (measured by QCM). (a) MI-Ni_3S_2, (b) H_2-Ni_3S_2, and (c) Ni_3S_2.

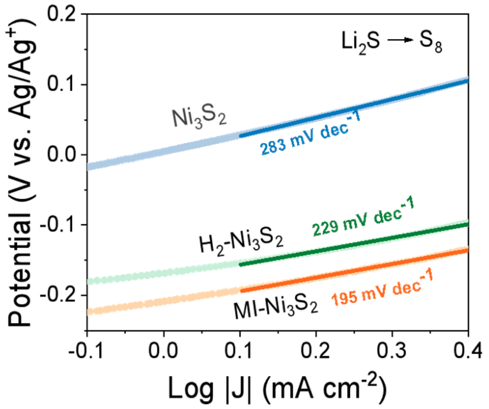

Fig. S18 The fitted Tafel plots corresponding to LSV curves with Ni_3S_2, MI-Ni_3S_2, and H_2-Ni_3S_2 electrodes.

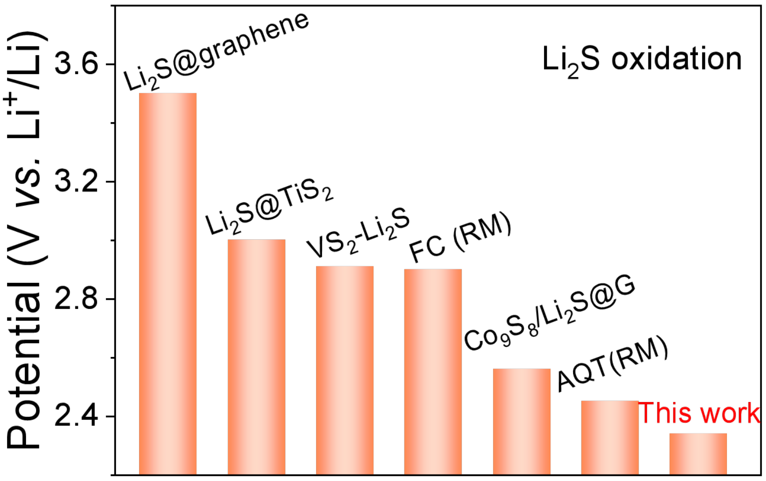

Fig. S19 Performance comparison of the Li_2S oxidation potential in this work with other. reported Li_2S cathodes[9-13].

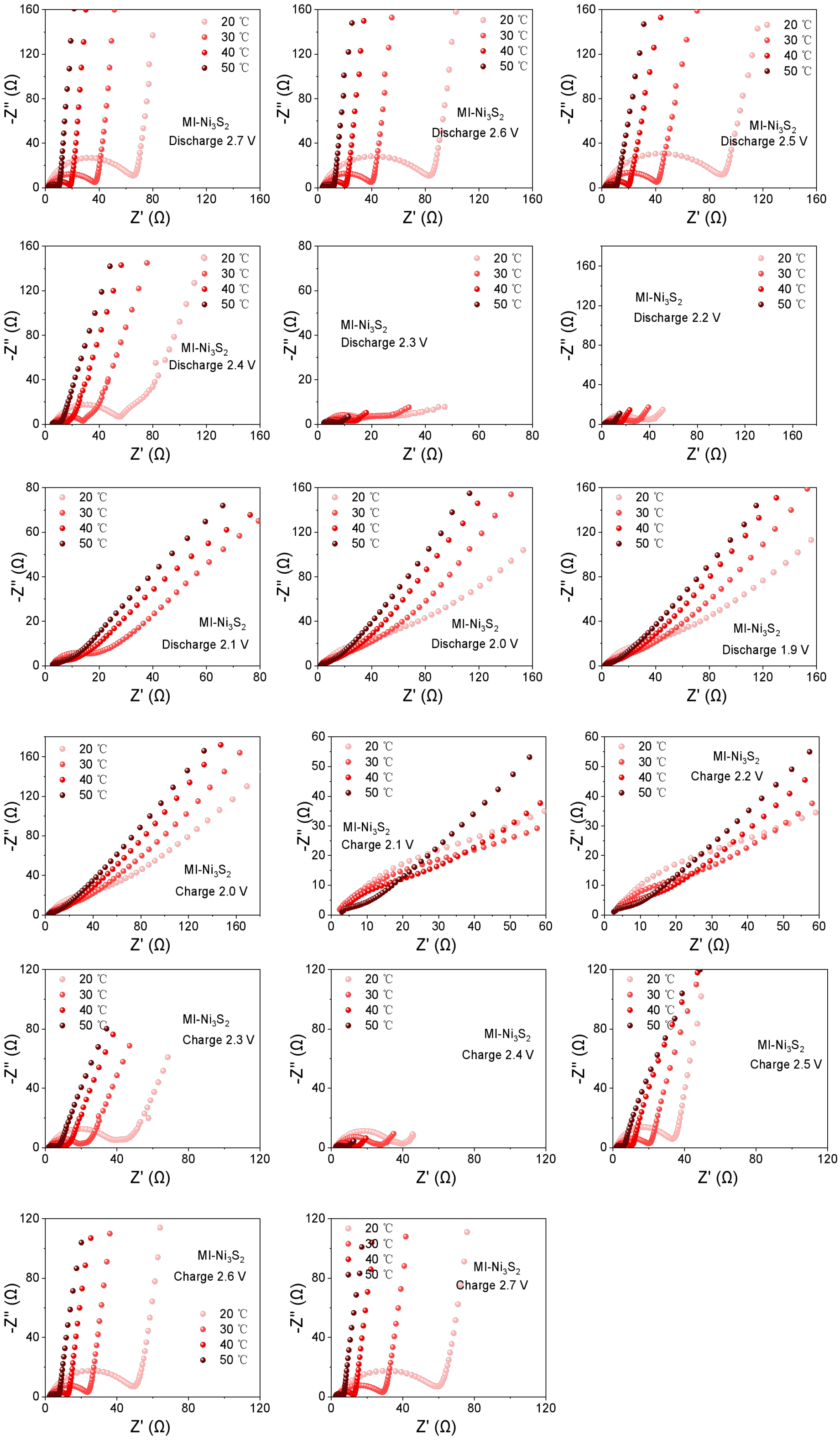

Fig. S20 EIS curves of Li-S cells with MI-Ni_3S_2 at different temperatures (discharge from 2.7 V to 1.7 V and then charge to 2.7 V).

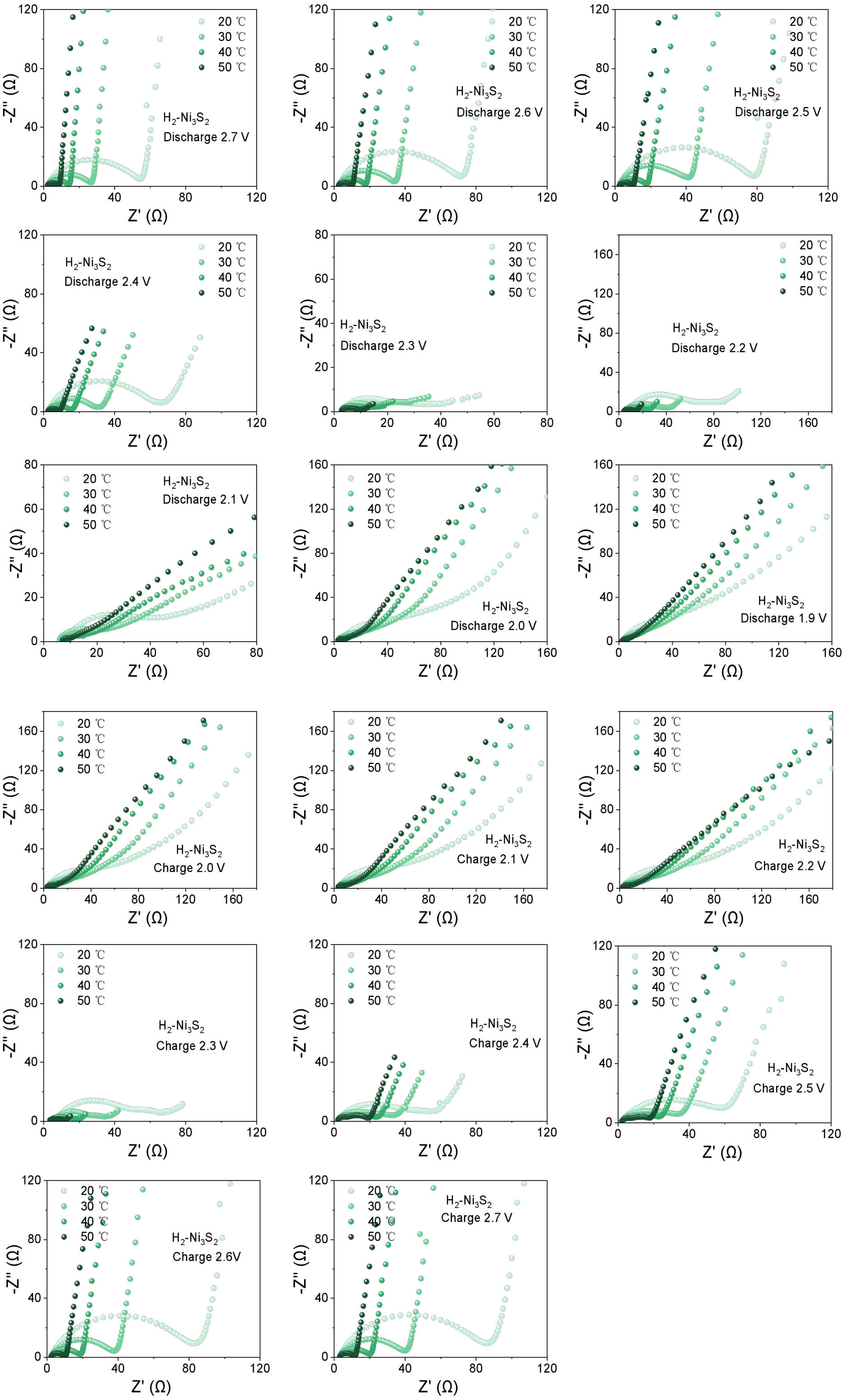

Fig. S21 EIS curves of Li-S cells with H_2-Ni_3S_2 at different temperatures (discharge from 2.7 V to 1.7 V and then charge to 2.7 V).

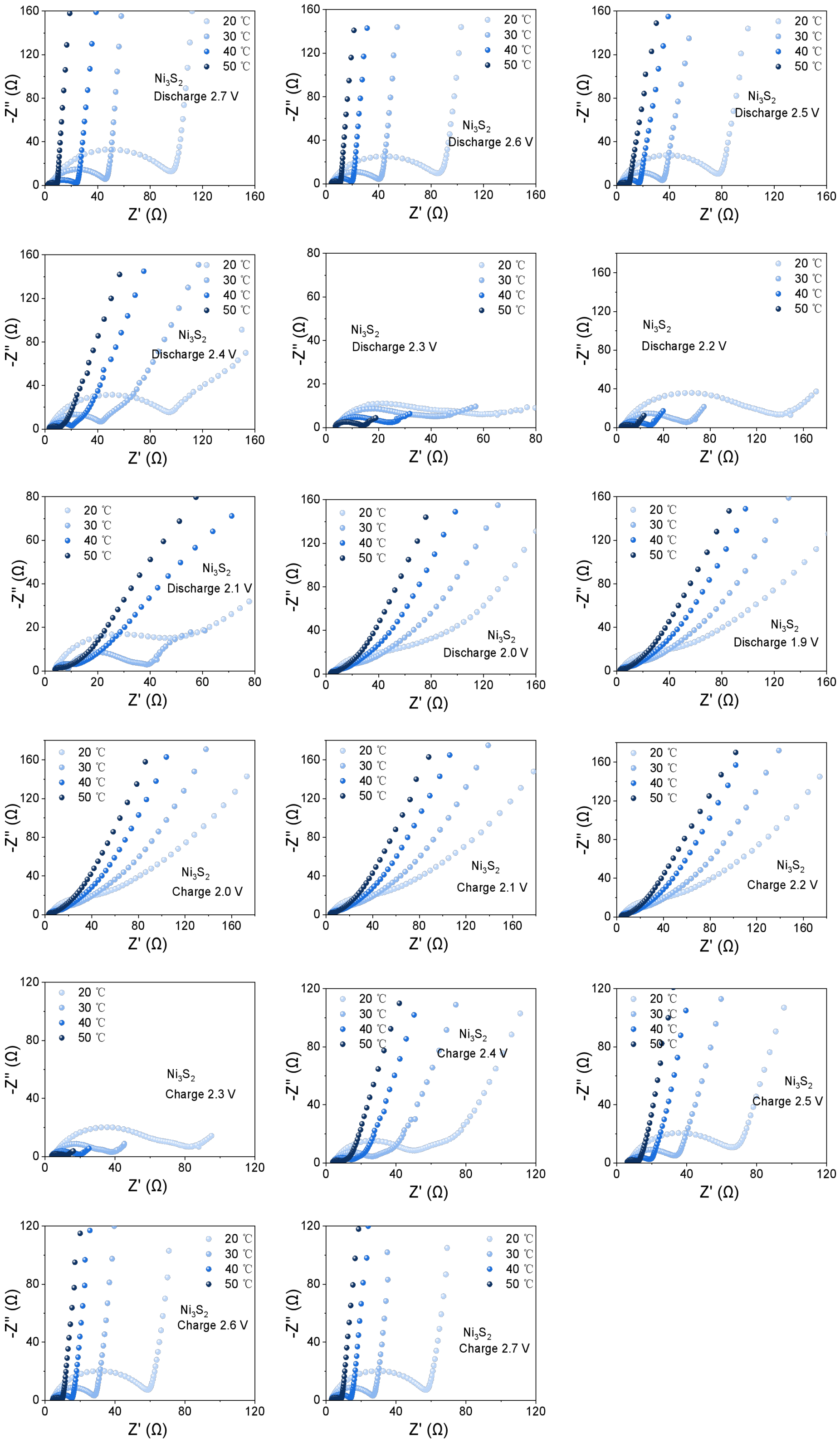

Fig. S22 EIS curves of Li-S cells with Ni_3S_2 at different temperatures (discharge from 2.7 V to 1.7 V and then charge to 2.7V)

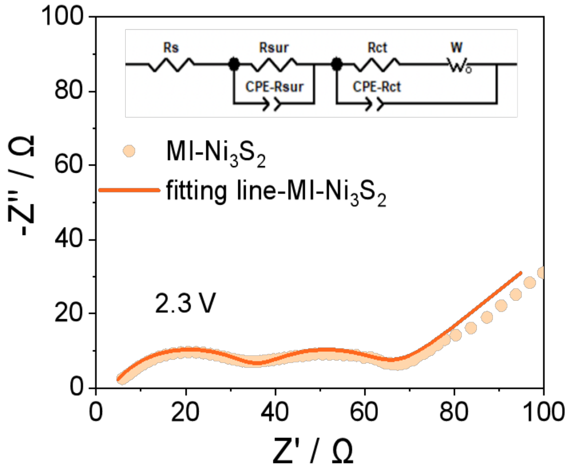

Fig. S23 The EIS curve and the corresponding equivalent circuit of the cells with MI-Ni_3S_2 at 2.3 V.

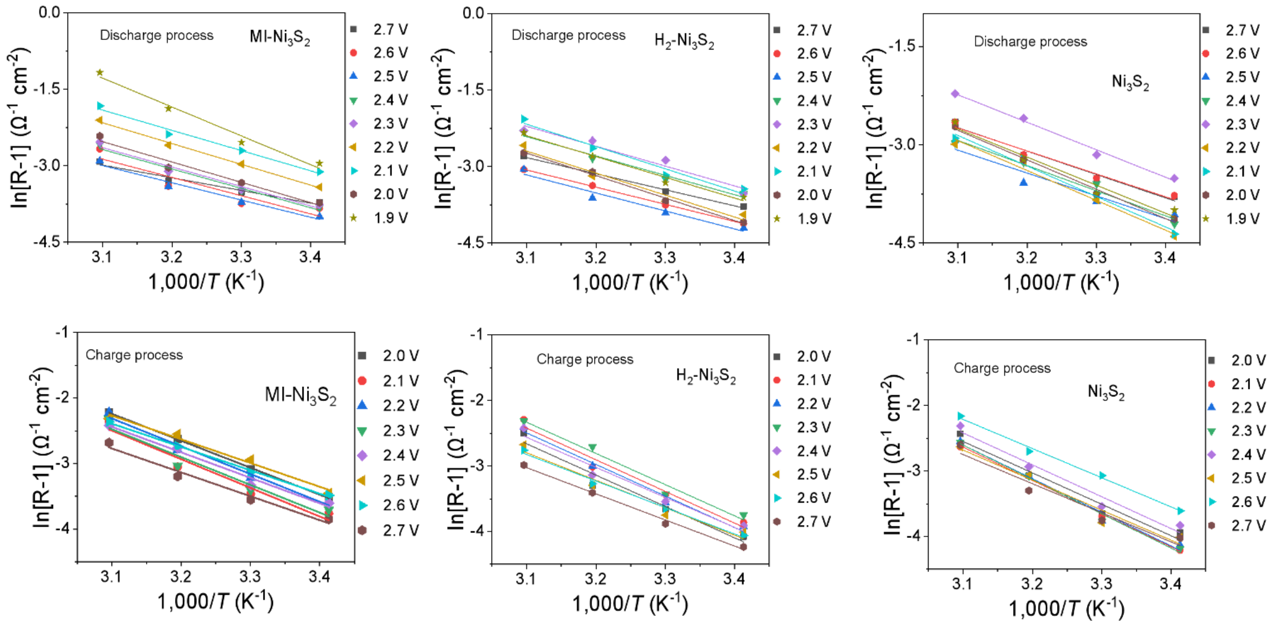

Fig. S24 The Arrhenius plots of Li-S cells with various catalysts during discharging and charging calculated by EIS curves.

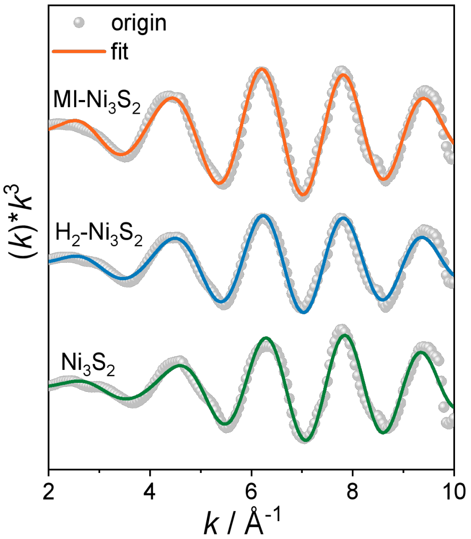

Fig. S25 The K space and the Fourier transform (FT) k3-weighted χ(k)-function of the EXAFS. spectra.

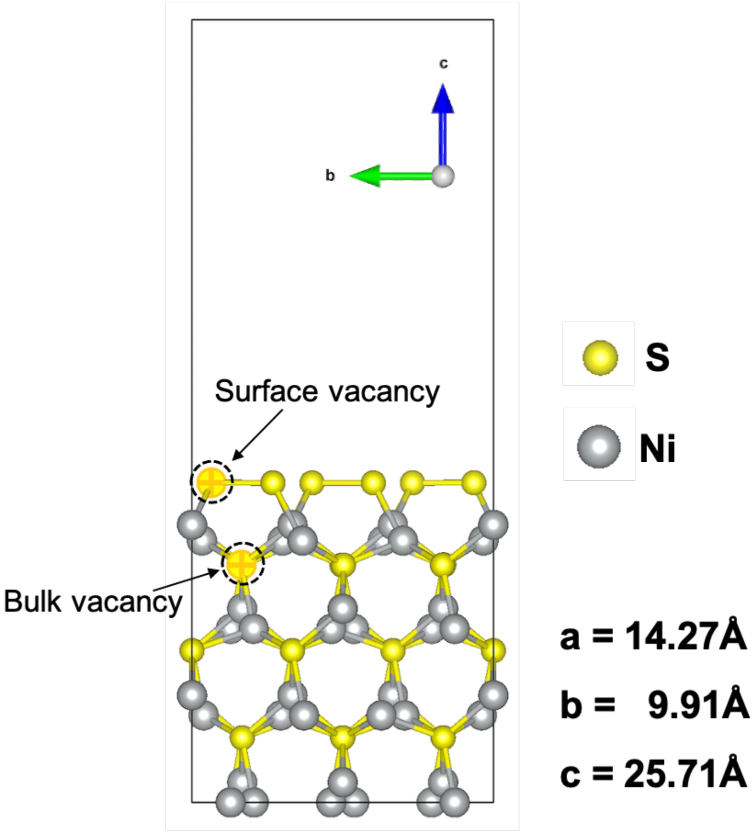

Fig. S26 The (110) slab model for Ni_3S_2 with bulk and surface sulfur vacancies.

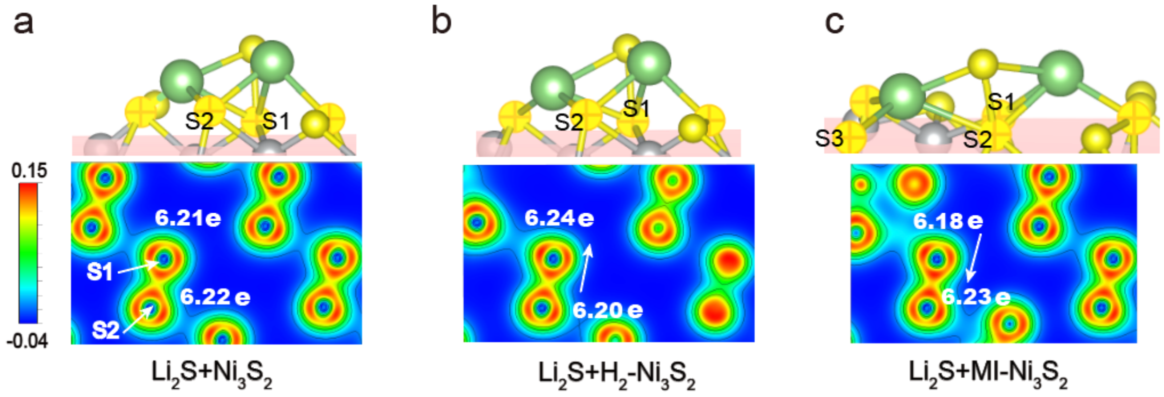

Fig. S27 Side view of the configuration diagram for Li_2S adsorbed on various catalyst surfaces. and the charge distribution of the active sites. (a) Li_2S with Ni_3S_2, (b) Li_2S with H_2-Ni_3S_2, and (c) Li_2S with MI-Ni_3S_2.

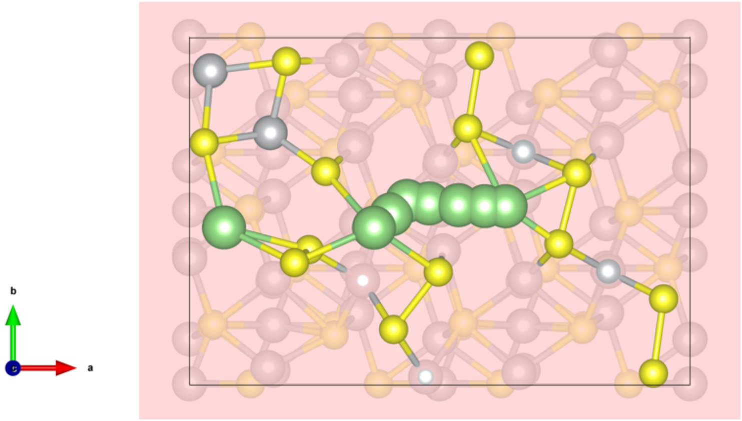

Fig. S28 Top view schematic representations of the decomposition pathways of Li_2S on MI-Ni_3S_2.

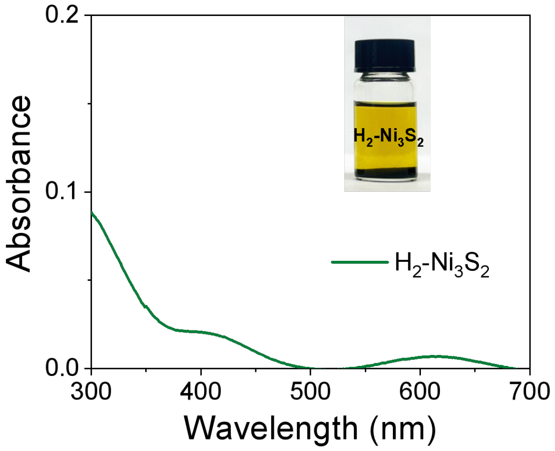

Fig. S29 The UV-visible absorption spectrum of Li_2S_6/DME solution with H_2-Ni_3S_2 after adsorption.

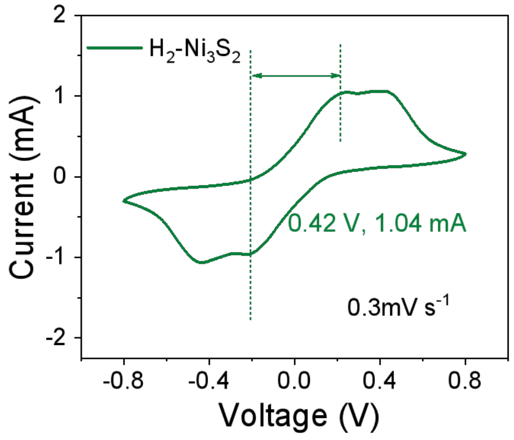

Fig. S30 CV curve of symmetric cell with H_2-Ni_3S_2.

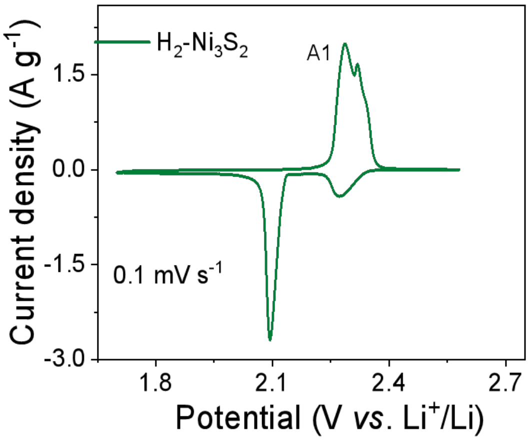

Fig. S31 The second cycle CV curve of the Li-S cell with H_2-Ni_3S_2 at a scan rate of 0.1 mV s^{-1}

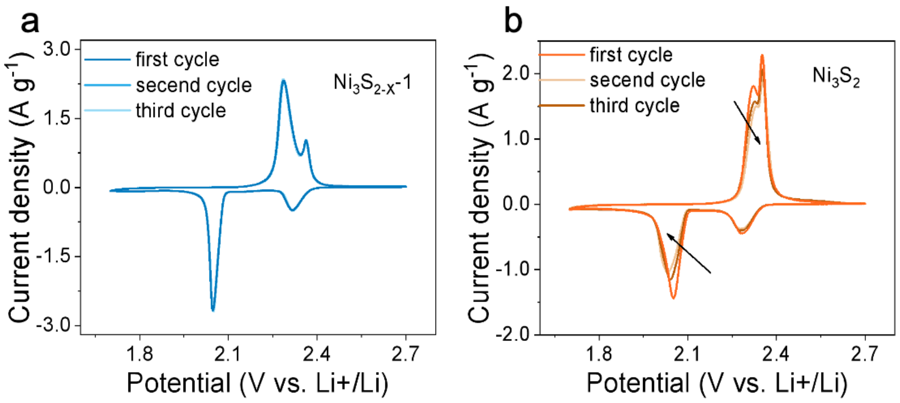

Fig. S32 CV curves of the Li-S cells with Ni_3S_2 and MI-Ni_3S_2 for the first three cycles. (a) MI-Ni_3S_2, (b) Ni_3S_2

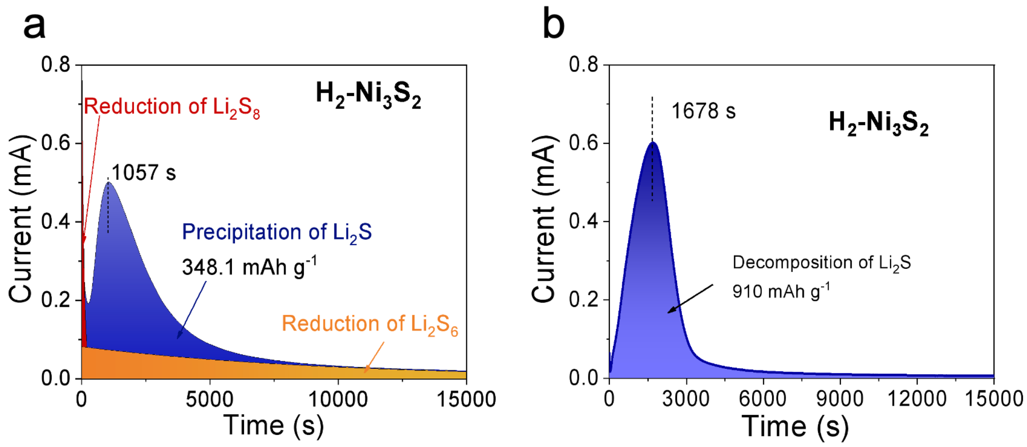

Fig. S33 The Li_2S deposition and dissolution measurements on H_2-Ni_3S_2/CP electrode. (a) Li_2S deposition, (b) Li_2S dissolution.

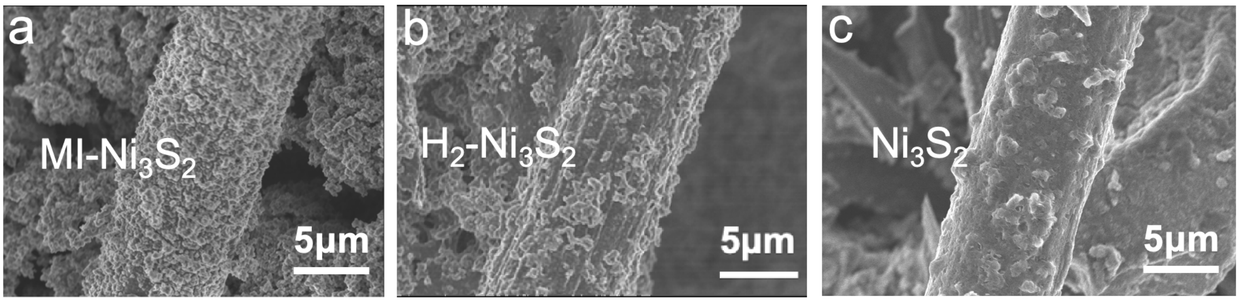

Fig. S34 The morphology of Li_2S deposition. (a) MI-Ni_3S_2/CP, (b) H_2-Ni_3S_2/CP, and (c) Ni_3S_2/CP.

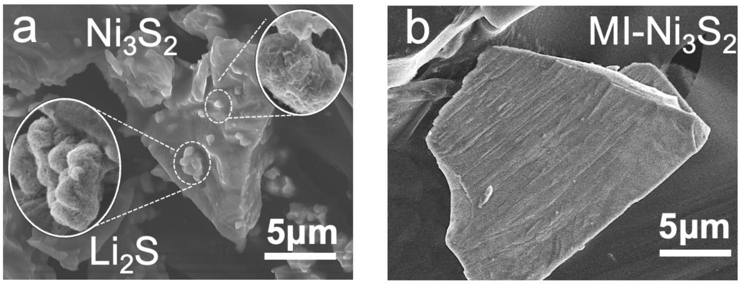

Fig. S35 The morphology of (a) Ni_3S_2/CP and (b) MI-Ni_3S_2/CP electrodes after charging.

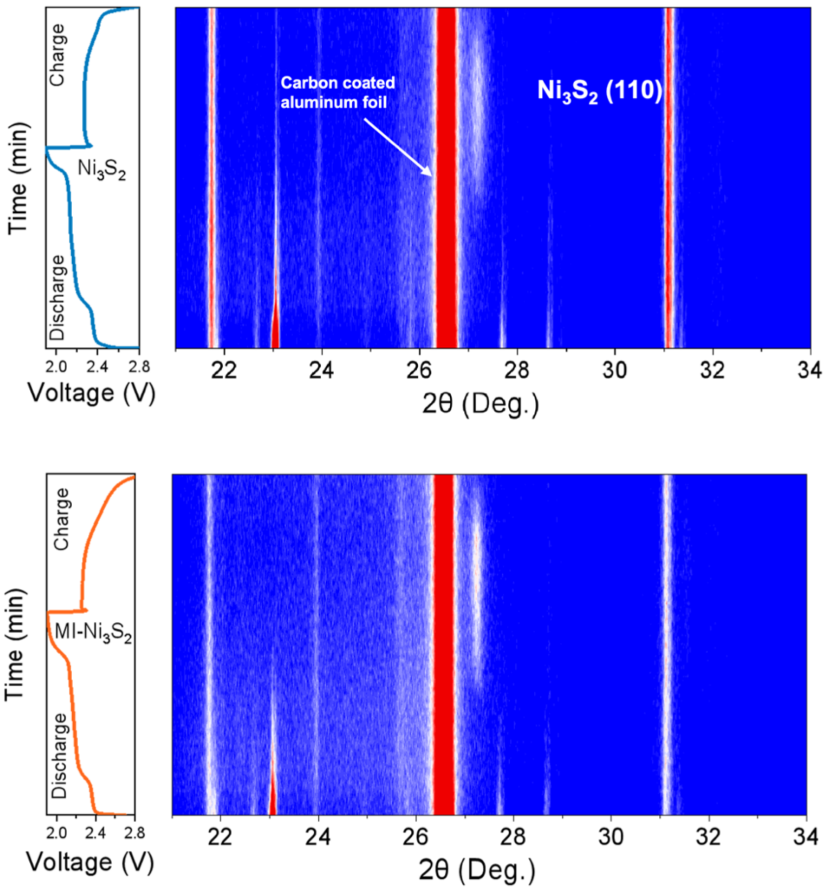

Fig. S36 The *in-situ* XRD patterns of Li-S cells with Ni_3S_2 and MI-Ni_3S_2 from 21°-34° (2θ) with the corresponding galvanostatic discharge-charge profiles.

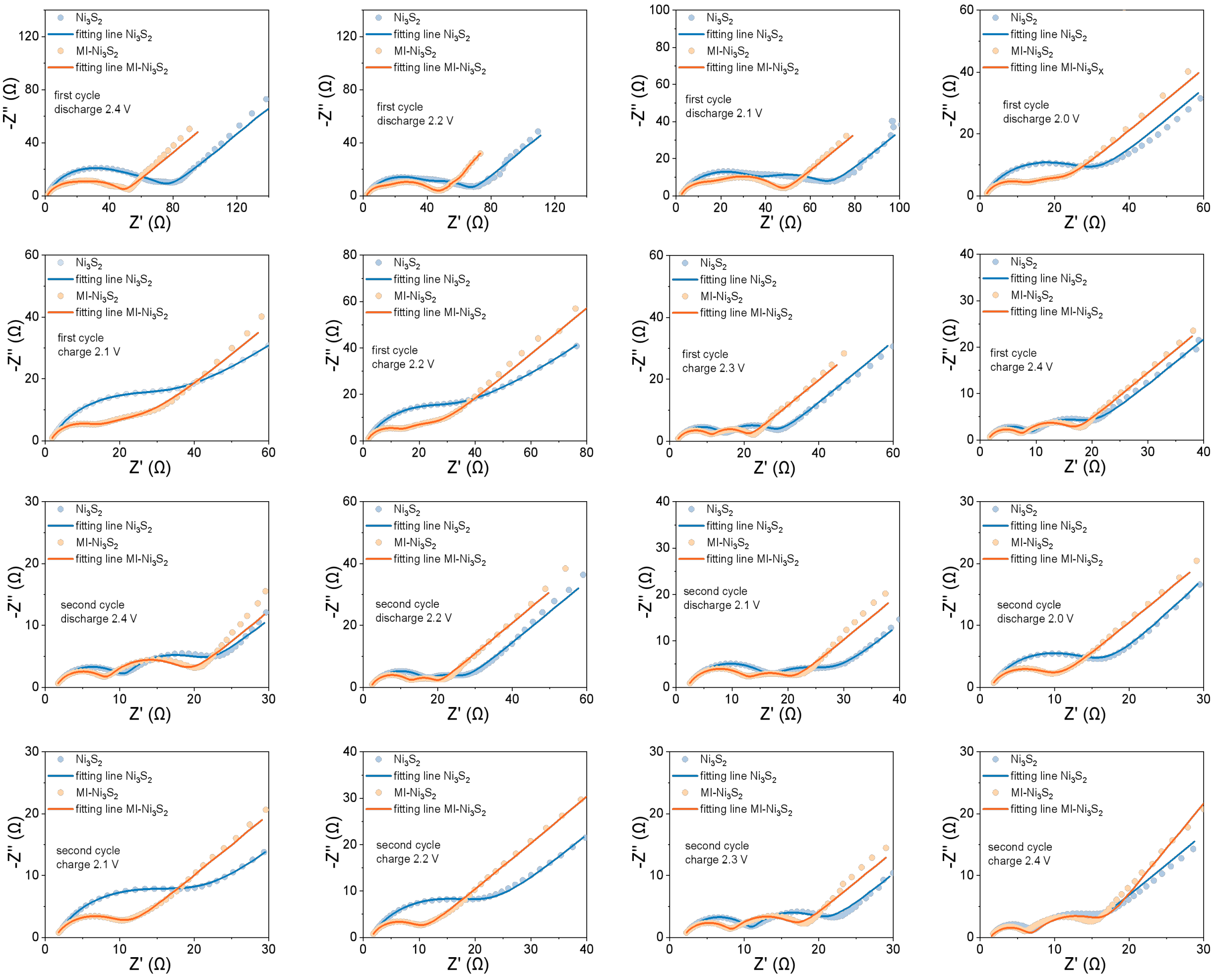

Fig. S37 EIS curves and fitting results of Li-S cells with Ni_3S_2 and MI-Ni_3S_2 at the given voltage during the whole discharge and charge process in two cycles (OCP-1.8-2.6V).

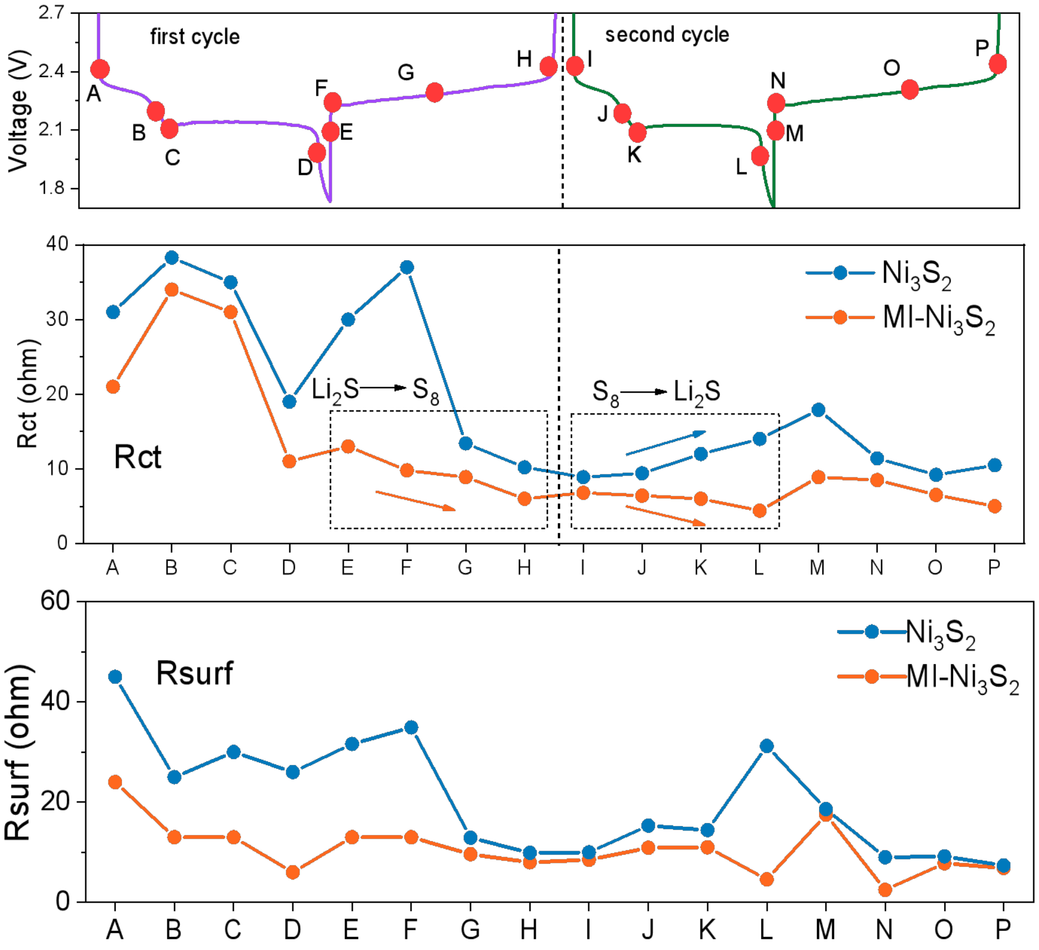

Fig. S38 Plots of R_{ct} and R_{surf} against the different potentials of discharge and charge in two cycles in the cells with Ni_3S_2 and MI-Ni_3S_2.

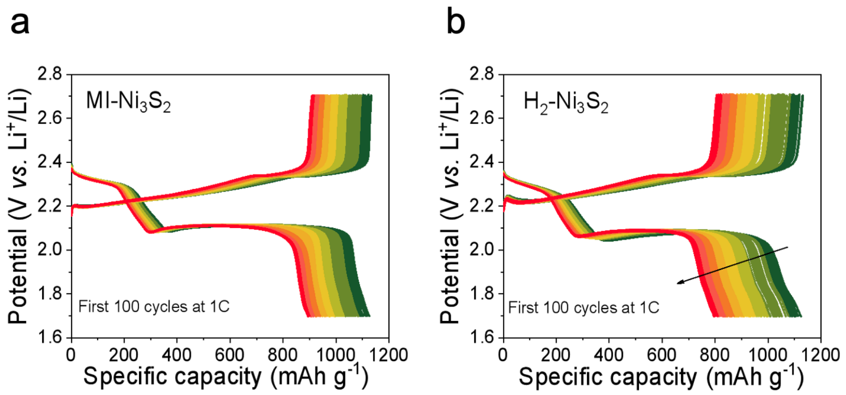

Fig. S39 Galvanostatic discharge-charge profiles at 1C with different cathodes. (a) MI-Ni_3S_2 and (b) H_2-Ni_3S_2 during the first 100 cycles.

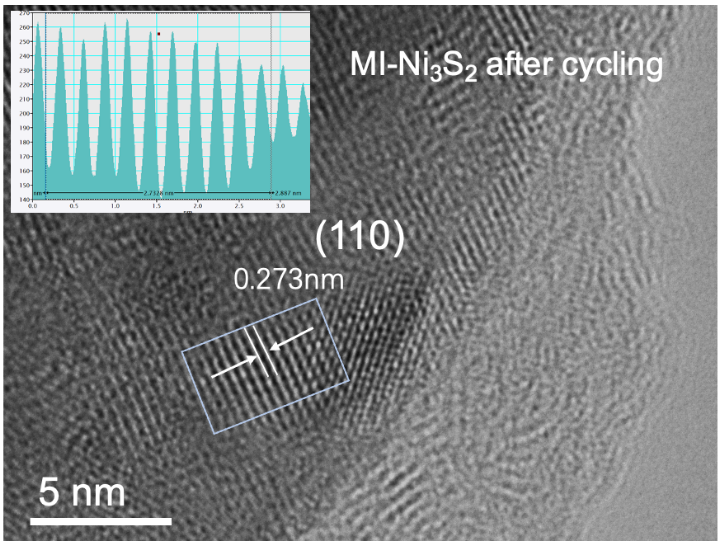

Fig. S40 HRTEM image of MI-Ni_3S_2 in cathode after cycling in Li-S battery.

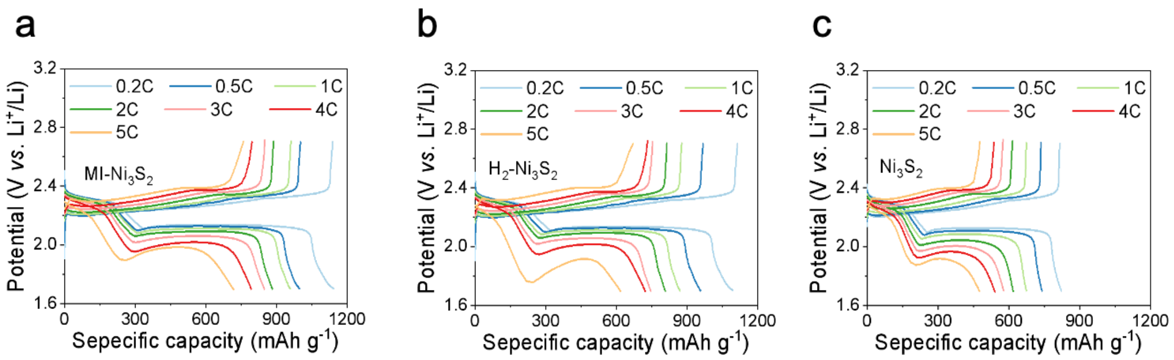

Fig. S41 Galvanostatic discharge-charge profiles at different rates with various catalysts. (a) MI-Ni_3S_2, (b) H_2-Ni_3S_2, and (c) Ni_3S_2.

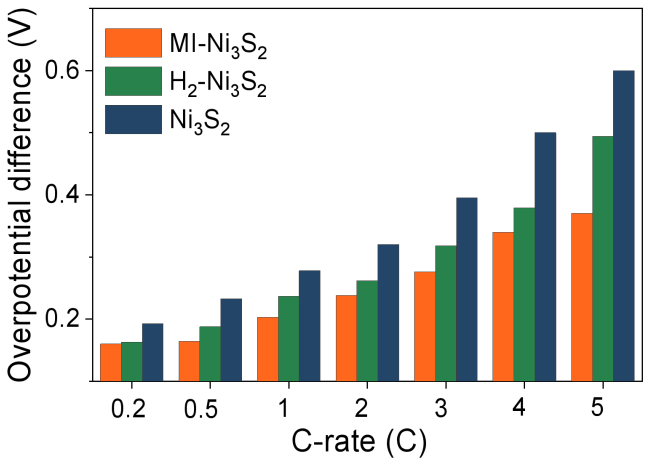

Fig. S42 Overpotential differences of batteries with various catalysts at different rates.

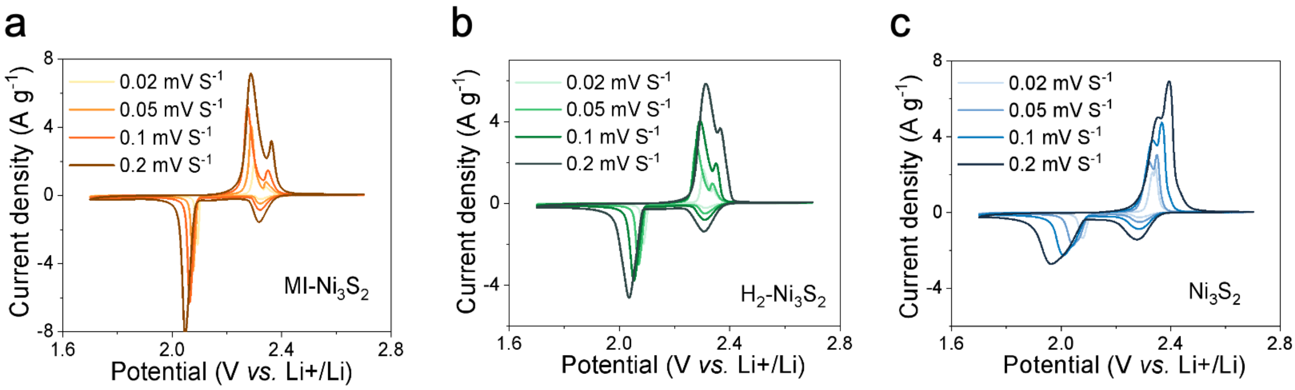

Fig. S43 CV curves of the Li-S cells with different catalysts at 0.02 mV s^{-1} to 0.2 mV s^{-1}. (a) MI-Ni_3S_2, (b) H_2-Ni_3S_2, and (c) Ni_3S_2.

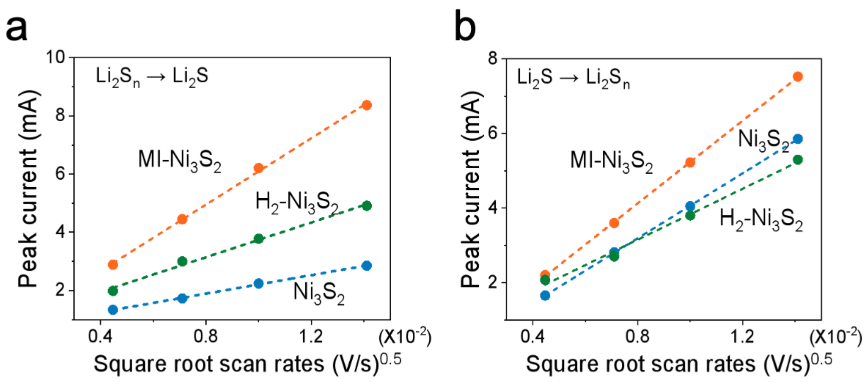

Fig. S44 Plots of I_p vs. $v^{0.5}$ of the cells with Ni_3S_2, MI-Ni_3S_2, and H_2-Ni_3S_2. (a) $Li_2S_n \rightarrow Li_2S$, (b) $Li_2S \rightarrow Li_2S_n$.

Note: The Li-ions diffusion rate (D_{Li}) is evaluated using the Randles–Sevcik equation, as below:

$$I_p = 2.69 \times 10^5 n^{1.5} A D_{Li}^{0.5} v^{0.5} C_{Li}$$

Where I_p is the peak current, n indicates the number of electrons in the reaction (n=2), A is the electrode area, and D_{Li} is the Li ions diffusion coefficient, C_{Li} is the concentration of Li ions in the positive electrode v is the voltage scanning rate.

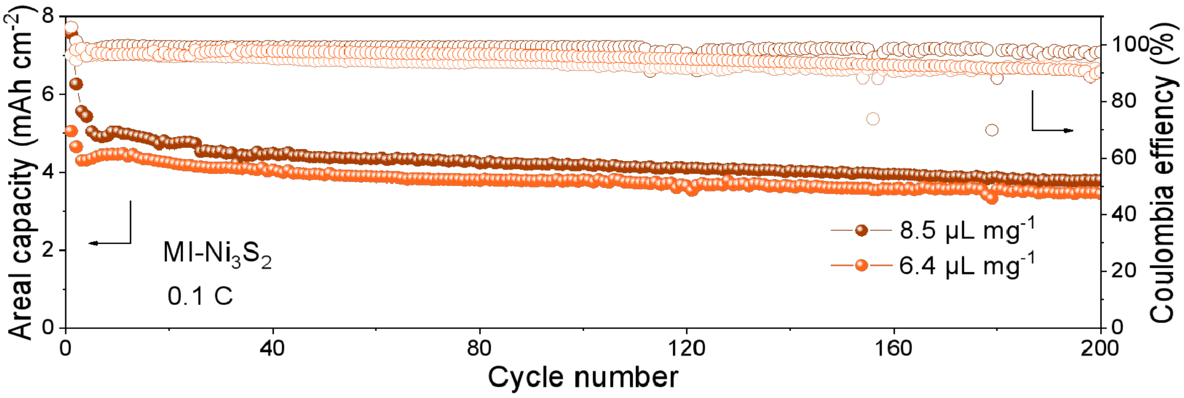

Fig. S45 Cycling performance of batteries with MI-Ni_3S_2 at different E/S ratios (0.1 C).

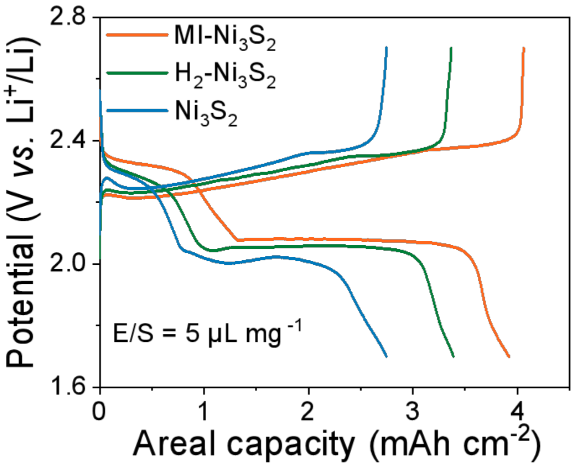

Fig. S46 Galvanostatic discharge-charge profiles at E/S ratio of 5.0 μL mg_S^{-1} with different. catalysts.

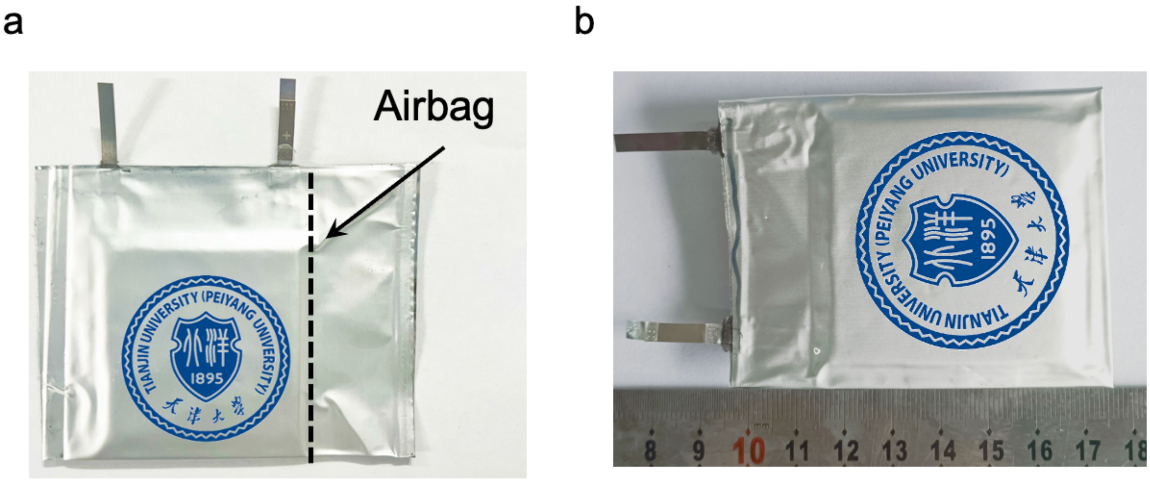

Fig. S47 The photos of assembled Li-S pouch cell (a) with and (b) without airbag.

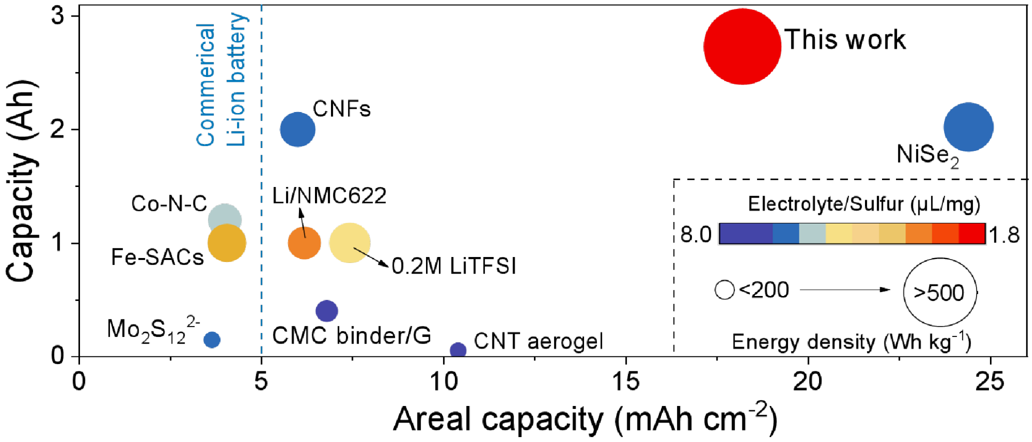

Fig. S48 Performance comparison between this work and previously reported Li-S pouch cells.

[14-19]

Table S1 The content and ratio of metal and S in various MI-MSs and MSs obtained by ICP-MS.

MS	S (at %)	M (at %)	MI-MS	S (at %)	M (at %)
FeS_2	64.07	35.92	MI-FeS_2	60.36	50.83
CoS_2	66.99	34.01	MI-CoS_2	59.46	40.54
Ni_3S_2	42.20	57.80	MI- Ni_3S_2	68.63	31.37
CuS	45.45	54.55	MI-CuS	44.83	55.17

Table S2 The content and ratio of metal and S in MI-Ni_3S_2(1-10) and MS obtained by ICP-MS .

Name	Ni_3S_2	MI-Ni_3S_2(1)	MI-Ni_3S_2(2)	MI-Ni_3S_2(5)	MI-Ni_3S_2(10)
Ni (at %)	57.80	68.63	63.58	61.85	59.70
S (at %)	42.20	31.37	36.42	38.15	40.30

Table S3 Calculation parameters of the saturated adsorption capacity of three catalysts for Li_2S.

	Li_2S concentration (mmol)	Mass change($\mu g/cm^2$)	c (μg/ml)	Mass of catalyst (μg)	Γ (ng/μg)	c/Γ
**MI-Ni_3S_2**	0.05	0.930	1.37	65	14.09	0.097
	0.03	0.720	0.66	66	10.90	0.061
	0.01	0.279	0.18	56	4.98	0.036
	0.005	0.140	0.09	55	2.55	0.032
**H_2-Ni_3S_2**	0.05	0.365	1.94	69	5.29	0.366
	0.03	0.273	1.11	59	4.63	0.264
	0.01	0.143	0.32	68	2.04	0.157
	0.005	0.095	0.13	69	1.44	0.094
**Ni_3S_2**	0.05	0.252	2.08	85	2.92	0.712
	0.03	0.151	1.23	55	2.72	0.452
	0.01	0.124	0.34	69	1.73	0.197
	0.005	0.090	0.140	68	1.32	0.106

Note: The saturated adsorption capacity is based on the QCM test, and the volume of the controlled solvent (methanol) is 1mL. Detailed calculation parameters are shown in Table S3.

Table S4 Ni-S bond length fitted by Fourier transformed (FT) with K space curvers of the EXAFS spectra.

Sample	n	s02	Sigma^2	e0	delr	Reff	R
**MI-NI_3S_2**	1.000	2.371	0.00319	-2.894	-0.01488	2.25190	2.2370
**H_2-NI_3S_2**	1.000	1.845	0.00111	-1.528	-0.02660	2.2519	2.2253
**NI_3S_2**	1.000	2.247	0.00377	-1.509	-0.02052	2.26140	2.2409

Table S5 The distance between the active site on the surface in different catalysts and the Li atom of Li_2S.

Catalyst	Li-S3(Å)	Li-S1(Å)
Ni_3S_2	4.09	3.70
H_2-Ni_3S_2	3.91	2.56
MI-Ni_3S_2	2.92	3.41

Table S6 The mass for each component in the Ah-level Li-S pouch cells.

Component Mass (g)	No.1	No.2	No.3
Cathode	2.31	3.32	3.91
Anode	1.00	1.17	1.17
Separator	0.91	1.07	1.07
Electrolyte	3.29	2.99	3.77
Al plastic film	1.30	1.30	1.30
Cell-pole and tap	0.30	0.30	0.30
Total	9.11	10.15	11.52

Note: The following equation was used to calculate the specific energy density of the pouch cell:

$$Eg = \frac{VC}{\Sigma \mathrm{mi}}$$

Eg is the energy density (Wh kg^{-1}), *V* is approximately 2.1 V for the Li-S pouch cell, *C* is the. discharge capacity (Ah), and mi is the mass for each component in the pouch cell (g). The total weight includes the weight of the cathode (sulfur, conductive CNTs, binder, Carbonized aluminum foil collects fluid and catalyst), anode (100 μm Li foil), separator, electrolyte (0.98 g mL^{-1}), Al plastic film, cell-pole, and tap. The cathode size is 5×5 cm^2, the negative electrode size is 5.5×5.5 cm^2, and the pole position is 1×1 cm^2. The specific energy density of the assembled sulfur pouch cell with five layers-cathode is 311 Wh kg^{-1}, and the six layers-cathode is 405 Wh kg^{-1} and 502 Wh kg^{-1}.

References

1. Zheng C, Niu S, Lv W *et al.* Propelling polysulfides transformation for high-rate and long-life lithium–sulfur batteries. *Nano Energy* 2017; **33**: 306-312.
2. Peng L, Wei Z, Wan C *et al.* A fundamental look at electrocatalytic sulfur reduction reaction. *Nat Catal* 2020; **3**: 762-70.
3. Yao W, Zheng W, Xu J *et al.* ZnS-SnS@NC Heterostructure as Robust Lithiophilicity and Sulfiphilicity Mediator toward High-Rate and Long-Life Lithium-Sulfur Batteries. *ACS Nano* 2021; **15**: 7114-30.
4. Wang R, Luo C, Wang T *et al.* Bidirectional catalysts for liquid-solid redox conversion in lithium-sulfur batteries. *Adv Mater* 2020; **32**: 2000315.
5. Hohenberg P, Kohn W Inhomogeneous Electron Gas. *Phys Rev* 1964; **136**: B864-71.
6. Perdew, Burke, Ernzerh of Generalized Gradient Approximation Made Simple. *Phys Rev Lett* 1996; **77**: 3865-68.
7. Kresse G, Joubert D, From ultrasoft pseudopotentials to the projector augmented-wave method. *Phys Rev B* 1999; **59**: 1758-75.
8. Grimme S, Antony J, Ehrlich S *et al.* A consistent and accurate ab initio parametrization of density functional dispersion correction (DFT-D) for the 94 elements H-Pu. *J Chem Phys* 2010; **132**: 154104.
9. Li Z, Luo C, Zhang S *et al.* Co-recrystallization induced self-catalytic Li_2S cathode fully interfaced with sulfide catalyst toward a high-performance lithium-free sulfur battery. *InfoMat* 2022; **4**: e12361.
10. Gao X, Zheng X, Tsao Y *et al.* All-solid-state lithium-sulfur batteries enhanced by redox mediators. *J Am Chem Soc* 2021; **143**: 18188-95.
11. Tsao Y, Lee M, Miller E C *et al.* Designing a quinone-based redox mediator to facilitate Li_2S oxidation in Li-S Batteries. *Joule* 2019; **3**: 872-84.
12. Tan G, Xu R, Xing Z *et al.* Burning lithium in CS_2 for high-performing compact Li_2S-graphene nanocapsules for Li-S batteries. *Nat Energy* 2017; **2**: 17090.
13. Zhou G, Tian H, Jin Y *et al.* Catalytic oxidation of Li_2S on the surface of metal sulfides for Li-S batteries. *Proc Natl Acad Sci U S A* 2017; **114**: 840-45.
14. Lim W-G, Park C-Y, Jung H *et al.* Cooperative electronic structure modulator of Fe single-atom electrocatalyst for high energy and long cycle Li-S pouch Cell. *Adv Mater* 2023; **35**: 2208999.
15. Fang Z, Luo Y, Wu H *et al.* Mesoporous carbon nanotube aerogel-sulfur cathodes: A strategy to achieve ultrahigh areal capacity for lithium-sulfur batteries via capillary action. *Carbon* 2020; **166**: 183-92.
16. Zhao M, Li B-Q, Chen X *et al.* Redox comediation with organopolysulfides in working lithium-sulfur batteries. *Chem* 2020; **6**: 3297-311.
17. Shi L L, Bak S M, Shadike Z *et al.* Reaction heterogeneity in practical high-energy lithium-sulfur pouch cells. *Energy Environ Sci* 2020; **13**: 3620-32.
18. Zhao C-X, Li X-Y, Zhao M *et al.* Semi-immobilized molecular electrocatalysts for high-performance lithium-sulfur batteries. *J Am Chem Soc* 2021; **143**: 19865-72.
19. Huang Y, Shaibani M, Gamot T D *et al.* A saccharide-based binder for efficient polysulfide regulations in Li-S batteries. *Nat Commun* 2021; **12**: 5375.
